# Scaling Laws in City Growth: Setting Limitations with Self-Organizing Maps

**DOI:** 10.1371/journal.pone.0168753

**Published:** 2016-12-22

**Authors:** Krzysztof Cebrat, Maciej Sobczyński

**Affiliations:** 1 Faculty of Architecture, Wrocław University of Technology, Wrocław, Poland; 2 Faculty of Biotechnology, University of Wrocław, Wrocław, Poland; Beihang University, CHINA

## Abstract

Do scaling relations always provide the means to anticipate the relationships between the size of cities, costs of maintenance, and the socio-economic benefits resulting from their growth? Scaling laws are considered a universal principle that describes the development of complex systems such as cities. It seems that regardless of their location or history, the growth of cities is associated with the super-linear or sublinear scaling of features such as the amount of space required, infrastructure, or human activities. However, the results of our research, based on grouping by Self-Organizing Maps, reveal some limitations in the application of scaling laws: the trends of urban growth behave in a different manner when we consider both a large and diverse collection of cities and a subset of cities alike. This finding complements the existing body of knowledge on the growth of cities and allows for a more accurate prediction of their future.

## Introduction

Scaling laws have been present in the debate on urban development and in the accompanying research since around 2006–2007 [1,2]. These developments were preceded by research and progress on the laws of developing networks [3].

A subsequent analysis of the statistical data supports the hypothesis that the number of important characteristics that describe a city along with its growth (number of inhabitants) varies exponentially; with an exponent greater than 1 in the case of, for instance, earnings and the number of innovations, and less than 1 when describing the supporting infrastructure [4,2].

Since then, many studies have focused on similar dependencies: network connectivity [5], urban supply networks [1], energy consumption [6], expansion of urban land uptake [7], pollution [8], and human interactions [9]. Observations were made regarding a similar level of accuracy in the spatial distribution of building geometries [10] and green space coverage in cities [11]. This does not conclude in any way the list of research and observations showing that, in spite of the diversity of their forms, cities are very similar in terms of their dynamics [2]. A number of existing extensive data sets confirm this hypothesis, showing that the benefits of growth and size exceed the costs that cities must bear. It is pointed out that this knowledge serves an important purpose from the point of view of urban planning [12].

However, studying a large and diverse group of cities may lead to the omission of the specific causes of globally-applicable rules [13].

Accordingly, the following is proposed: if scaling laws are valid for entire urban systems, they should also be considered important locally—not just in the consideration of cities from more than one country or region (this already has been done, for example, for the United States, Germany, China, Korea, Mexico, etc.), but within the boundaries of one country looking at groups of cities demarcated by their similarities.

Cities in different geographic areas at different times [4] obtain different exponent values for different features. But the question remains: will the relationships and trends that come to light when considering all of the cities of a particular region, without the criteria for selecting a sample, be the same when one analyzes internally-similar groups?

Our research shows that they are not, and this may serve as the basis for a broader discussion on the causes of the scaling laws lying within the boundaries of cities.

## Describing the Cities

The study involved 881 of the current (2016) 915 Polish cities (towns with municipal rights), regardless of their size, within the administrative borders adopted by the Central Statistical Office.

The study excluded only those cities that have received municipal rights during the audited period or at a later date, or those without consistent statistical data.

Due to administrative changes, the studies covered the cities during the years 2002–2012. For Poland, this period was marked by a period of rapid development, which likely has an impact on the results of the studies.

The method of determining the groups from a larger set of cities had to be objective and based on many data points, but with no assumptions made on the nature of the subsets (size, number, characteristics) and correlations between the data.

Although some other techniques of examining multidimensional data exist (such as deep learning theory [14]), it was found that Kohonen’s Self-Organizing Map (SOM) would serve as the best tool for identifying groups of cities similar to each other while satisfying the mentioned requirements [15]. It enables the unsupervised learning process of a network and the mapping of multidimensional data and dependencies onto a smaller number of dimensions.

It was decided that the indicators related to the metabolism of the city will consist of features describing the city itself, and that they will serve (on the whole) as the basis for their subsequent grouping.

Increasing population, area, diversity, and multiplicity of connections allow cities to increase the speed and efficiency of the acquisition, processing, and—ultimately—dissipation of energy, which comprise the basis of the city metabolism as researched for many years [16]. Therefore, this process is crucial from the point of view of urban development. Assumptions underlying the selection of features, as well as the features themselves, are discussed in detail in the Appendix in S1 File.

10 indicators (features of cities) were constructed. They were used to create a database describing city metabolism across 3 main sectors of the city: natural, economic, and housing [17]:

Feature 1 (F1): [factorized area of forests / city area]
Feature 2 (F2): [total usable floor space of (all) dwellings / city area]
Feature 3 (F3): [total income of city residents (in terms of energy intensity) / city area]
Feature 4 (F4): [budget expenditures of the local government / total income of city residents]
Feature 5 (F5): [budget expenditures per capita]
Feature 6 (F6): [number of enterprises / city area]
Feature 7 (F7): [number of enterprises per capita]
Feature 8 (F8): [Enterprise Diversity Index (EDI)]
Feature 9 (F9): [gas consumption in households / total usable floor space of dwellings]
Feature 10 (F10): [electricity consumption in households / total usable floor space of dwellings]

The database transferred for further analysis was kept anonymous, as the grouping was carried out solely on the basis of a set of characteristics. City names and their locations were encoded.

The sources of data are provided in the Appendix in S1 File.

## Grouping the Cities

Let *m*_*i*_, *i* = 1,…,881, be a town described by a real vector _1×110_**x**_*i*_ = [_1×11_**z**_*i*,1_, …, _1×11_**z**_*i*,*k* = 10_], where _1×11_**z**_*i*,*k*_ contains the variable *k* = 1, …, 10 measured in each of the 11 years (2002–2012). The set of all 881 towns forms a matrix _881×110_**A** = [_881×11_**Z**_1_ … _881×11_**Z**_*k* = 10_]. The aim is to group the towns, according to their similarities, on an array of nodes (*neurons*). This process, called *mapping*, was described by Kohonen [15] as an SOM algorithm. It converts complex relationships between high-dimensional data into simpler representations on one or two-dimensional arrays.

The basic assumption of SOM is that grouping is accomplished without any:

- a priori imposed requirements for the number of defined groups (neurons),
- variable weighting (opposite to ranking grouping),
- assumptions for correlation structure within data.

Neurons in Kohonen’s maps (based on the definition [15]) have a fixed neighborhood, what is very important in projecting multidimensional similarities into (a simple) perception map.

Initially, every node (neuron) of the map has a **random** set of co-ordinates (a reference vector selected at random from the domain of the input samples). Next a vector (input vector) **randomly chosen** from the training dataset (the uncompressed, original dataset) is presented to the array, and a node which one's co-ordinates are most like the input vector (the distance between them is smallest) is chosen (a Best Matching Unit—BMU). Than the radius of the neighborhood of the BMU is calculated. Nodes (neurons) found within this radius are neighbors of the BMU and their co-ordinates (reference vectors) are adjusted to make them more like the input vector. In two dimensional arrays every node (except for edge nodes) has four neighbors. The iterations are carried out until changes in the array are negligible.

Every input vector (town in this case) is presented to the array every iteration. Therefore the end result of mapping (assigning cities to neurons) does not depend on which vector starts the process.

The following parameters are adopted in the process of grouping cities:

- topology type: rectangular;
- learning rate function type: inverse;
- neighborhood function type: Gaussian;
- initial learning rate parameters, for the two training phases are: 0.05 and 0.02;
- running length in the two training phases: 2000 (ordering phase) and 8000 (fine-adjusting phase).

In particular, for every town, its input vector _1×*p*_**x**, is presented to every neuron on the array and a distance *d*(_1×*p*_**x**, _1×*p*_**w**) between _1×*p*_**x** and a reference vector _1×*p*_**w** connected to the neuron is computed. Next, the _1 ×*p*_**w** with the smallest distance, called the *best-matching neuron*, is modified and becomes more similar to the _1 ×*p*_**x**. After many iterations, each city is assigned to a neuron and is as similar as possible to the other towns of that neuron. We used the classic Euclidean distance *d* between the input vector and the reference vector *d* = ‖_1×*p*_**x**−_1×*p*_**w**‖_2_. Variables in the sub-matrix _881×11_**Z**_*k*_ are states of variable *k* in each of the 11 years studied and are highly-positively mutually correlated. For this reason, the data included in _1×11_**z**_*k*_ were reduced to a single value of the first principal component. In the case of *k* = 8 variables the first component explained at least 90% of the total variance; in case of the two others variables, it was 76% and 78%, respectively. As a result, data in the matrix _881×110_**A** were reduced to data in the matrix _881×*k* = 10_**B**.

The SOM algorithm creates a map that stores information maintaining any topological relationships within the training (original) dataset. There are no lateral connections between neurons within a map. A single neuron is a group of cities, with features very similar to each other. Adjacent neurons contain cities that differ a little (differ more than cities within a single neuron). The more distant neurons are, the bigger the differences between cities.

The authors chose the rectangular array as there are no crucial differences between two main kinds of topologies of a SOM (hexagonal and rectangular) (see [15], pages 109–110 and 159–161) in quality of preserving original multidimensional relationships among data.

Since mapping data with the SOM algorithm is an autonomous process—SOMs learn to classify data without supervision—one does not know at start which map will be best: the results of the clustering and thus the best topology are unknown. To choose the best topology of the network, we used the Bayesian information criterion *BIC* as a measure of compromise between the quantization error $e=\sum_{i=1}^{881}d_{i}$ and the number of neurons in the SOM. The second criterion was the so-called topological error $f=100\%\times \frac{1}{881}\sum_{i=1}^{881}t_{i}$ where for every *i*^*th*^ town *t*_*i*_ = *I*(**w**_*i*,1°,_ **w**_*i*,2°_) and *t* = 0 if the first best-match neuron **w**_*i*,1°_ and the second-best-match neuron **w**_*i*,2°_ are neighbors on the SOM, and *t* = 1 when they are not. These universal criterions are independent to any observed or expected final results.

We tested the topologies of 36 maps. To be more accurate—the maps were tested whether they represent the multidimensional data (ultimately 10 D data in this case) in a lower dimension space in a suitable manner. The best one, according to the *BIC*, had a dimension *x* = 1 × *y* = 12 neurons and *f* = 3.18%. Evaluation of topologies of all 36 maps, as well as the number of cities in the neurons and the average quantity of features, are presented in Tables A-C in S1 File.

## Results

The applied method of analysis allowed for the grouping of Polish cities into subsets with respect to the degree of similarity of all the features chosen as important; subsequently, it allowed for the determination of whether the exponents indicated by scaling laws and valid globally may also accurately describe the local relations presented in each group. The results indicate that they do not.

As mentioned earlier, cities in one group (neuron) are similar to each other in terms of all 10 features and in terms of the history of their changes.

By means of the conducted grouping, it is observed that cities in Poland vary considerably from each other, that these differences arise from the way in which the cities process energy, and last but not least, that the boundaries between these groups are not trivial. Clearly there is a close relationship between the size and wealth of a city and its membership in a particular group, but it may also be observed (based upon the composition of groups) that these are not the only factors determining the placement of the city within the network. There must be something else that causes cities as different as Warsaw (capital; over 1.7 mln. citizens), Opole (121,000 citizens) and Wieliczka (about 21,000 citizens) to be grouped together (in one neuron).

It may be considered that the grouping provides an image that shows the different development strategies chosen by Polish cities over the past 11 years. However, this was not the most important finding.

Grouping the cities also shows that the scaling laws “work” well for the collection of Polish cities as a whole, but the relationships become different if one considers groups of cities that are similar to each other.

The results are most interesting when comparing the “global” and “local” relationships between the size of the cities and the income of their residents.

The dependencies of power consumption, the number of economic activities, and the value of budget expenditures are available in the Appendix in S1 File.

**Fig 1:**
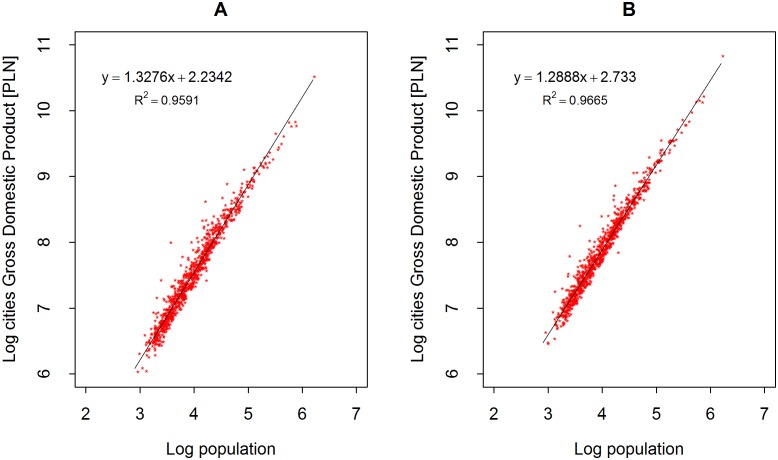
Scaling of GDP in all Polish cities (A) in 2002, and (B) in 2012.

The results of the analysis of the Gross Domestic Product (GDP) of a city in relation to the number of its inhabitants *N* (for all Polish cities) is shown in Fig 1. If the problem is considered across a large and diverse sample, one can see the validity of the scaling relation *GDP*(*N*) = *αN*^*β*^ with exponent *β* equal to *β* = 1.32 and *β* = 1.28 for the years 2002 and 2012, respectively, and an *R*^2^ = 0.96 (2002) and *R*^2^ = 0.97 (2012). In other words, when taking into account all of the studied cities, population growth is associated with a greater than proportional increase in the income of their residents. Interestingly, in these eleven turbulent years of Polish development, the exponent quite clearly decreased.

**Fig 2:**
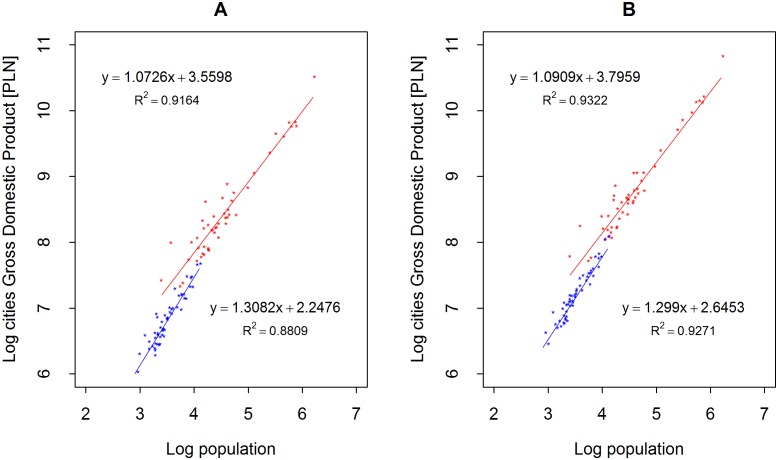
Scaling of GDP in cities grouped in neuron no.1 (red) and no. 12 (blue) in (A) in 2002, and (B) in 2012.

This changes (as shown in Fig 2) when one takes into account only the cities grouped into the first (no. 1) and last (no. 12.) neuron (groups of cities most different from each other; the cities in one neuron are similar to each other, while the cities in different neurons differ more and more as the neurons become more distant). Scaling of GDP in cities grouped in each neuron in year 2002 compared to year 2012 is shown in S7–S18 Figs.

The relationships are changing—and these changes are significant. The exponent remains at around *β* ≈ 1.3 for cities grouped in neuron 12, but the dependencies in the cities of neuron 1 are close to proportionality, i.e., *β* ≈ 1. This is even more surprising considering that the first neuron groups wealthy cities that, according to the analysis of the average values of characteristics, are also large in terms of their populations.

It appears that this finding should lead to a revision of the hypothesis that the scaling of the gross product of the size of cities is always super-linear [4]. In the present case, this is true only for small cities (the average population in neuron 12 is only 3,990 inhabitants).

The validity of this rule for cities outside of Poland requires further study, but it can be seen that small towns may, in some cases, serve as more attractive locations for investment in the so-called human resources.

**Fig 3:**
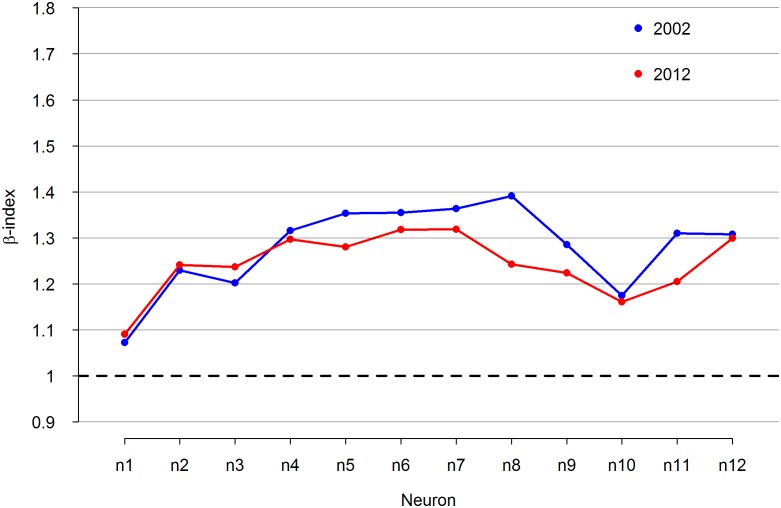
The *β* exponent of gross product/population scaling, in relationship to groups of cities.

In the cities of neuron 1, both the number of enterprises and the level of their diversity are greatest. These serve as measures of inhabitants’ social and economic activity. They are processes wherein many individuals interact with each other. In the case of such dynamic local interactions, one can expect the power law with exponent *β* ≅ 1 to be observed across many areas of human activity [18]. According to the results presented in Fig 2, we can conclude that cities grouped in neuron 1 are wealthy and have a higher number and a higher diversity of enterprises due to sufficiently high levels of social, cultural, political, and economical interactions between their inhabitants. It’s noteworthy that the SOM network was able to detect and to group such towns based on only a few global indicators. For the results presented in Figs 1 and 3, we must conclude that the cities grouped in neuron 1 are, unfortunately, nationwide exceptions. This is in keeping with the fact that Poland has one of the lowest levels of social capital in Europe. Unfortunately, higher confidence translates to economic benefits due to the lower costs of transactions, shorter time spent on negotiation and actualization, and the decreased necessity of monitoring during the completion of contractors. Higher social capital also leads to a higher probability of keeping of an agreement, which is a key stimulating factor for the growth of enterprise.

It should also be noted that in most groups of cities, throughout the 11 observed years the exponent decreased markedly (Fig 3). On one hand, this proves the existence of certain processes, which reduce the efficiency of the functioning of cities; on the other hand, this reduction also decreases the differences between various groups of cities. SOM helped to identify the cities that most contributed to these changes. In the case of Poland, the biggest change occurred in those grouped into neuron 8, many of which are holiday resorts.

## Conclusions and Discussion

Not only do cities of different sizes have very different properties [4], but these properties are best defined by different exponents in scaling relations. What’s more, scaling relations may not even always apply (see, e.g., budget expenditures on maintenance as shown in the Appendix in S1 File. Applying SOM gives hard evidence confirming findings of Strano and Sood [19].

The results show that the predictions of the characteristics of cities may be reliable only if a sufficiently large and diverse collection of cities is used. By means of an oft-used comparison, one could recognize that there are fairly general laws that apply both to anthills and beehives, and on this basis, one could be tempted to state that the same laws apply to both ants and bees [20]. Meanwhile, when one considers insects that are similar to each other (e.g., the family of bees together, the family of ants together, regardless of a particular species or habitat) the relationships between the important features defining the parameters of their lives are very different and not always subject to scaling laws.

This analogy perhaps best explains the results: we considered different groups of cities similar to each other, and we noticed that trends in the characteristics describing the cities contained within a group (neuron) never coincided with the trends exhibited by all of the cities taken together.

This applies in particular to the economic activity of inhabitants, energy consumption, and the costs of maintaining cities.

It should be emphasized that the cities forming a group were not arbitrarily chosen, but were assigned into groups as a result of the unsupervised learning process of a neural network, so the composition of the groups did not meet anyone’s preset expectations. A city was allocated to a particular group because it was similar to other members of the group in terms of some important characteristics.

One might also ask, whether there is a noticeable volume effect in the results of the β exponent. Since final groups contain different number of cities (ranging from 42 to 107) is it possible that differences in β arise from their volume? This could be easily explained by the algorithm of grouping: the exponent depends on the values of features characterizing cities in different groups, because those features are responsible for assigning cities to groups—and therefore for the volume of each group. Not the other way around. The volume of each group is a consequence of a process without any external supervision and a priori imposed requirements. The only thing one knows, and what really matters, is that every group (neuron) consists of cities similar to each other, and no matter how many cities are there in a group, they will still be similar.

Many questions arise from research findings. For instance, if one considers scaling relationships as an optimization problem as Bettencourt sees it [4], one then has to ask why large Polish cities manage this process distinctly worse than do mid-sized cities and towns.

Increasing costs of maintenance may be the answer, as analysis shows that the best fit (see S3 Fig)–performed using ordinary least squares minimization—is expressed by a second degree polynomial equation. This means that in relation to the population, expenses grow faster than incomes.

This could be also an effect of analyzing cities in their administrative borders adopted by the Polish Central Statistical Office, similarly as in case of Netherland cities discussed by van Raan, van der Meulen and Goedhart [21].

One may treat these findings as a very local phenomenon—that only in Poland are the relationships between the activities of its residents exhibited by super-linear scaling in some groups of cities and sublinear scaling in others; that rapid socio-economic changes are responsible for significant changes in the scaling exponents of groups of 3, 5, 8, and 11–12 cities over the past 11 years.

But, on the other hand, if scaling relationships maintain this level of diversity across the different groups of cities, the cognitive value of these relationships may only be related to a very general understanding of the properties of urban systems.

## Supporting Information

S1 Fig Scaling of electricity consumption in all Polish cities (A) in 2002, (B) in 2012, and the *β* exponent of electricity consumption scaling in relationship to groups of cities. (TIF)

S2 Fig Scaling of number of enterprises in all Polish cities (A) in 2002, (B) in 2012, and the *β* exponent of number of enterprises in relationship to groups of cities. (TIF)

S3 Fig Scaling of number of budget expenditures in all Polish cities (A) in 2002, (B) in 2012, and the *β* exponent of budget expenditures in relationship to groups of cities. (TIF)

S4 Fig Scaling of budget expenditures (2012) of cities grouped in neurons 1–4. (TIF)

S5 Fig Scaling of budget expenditures (2012) of cities grouped in neurons 5–8. (TIF)

S6 Fig Scaling of budget expenditures (2012) of cities grouped in neurons 9–12. (TIF)

S7 Fig Scaling of GDP in cities grouped in neuron no.1 in 2002 and in 2012. (TIF)

S8 Fig Scaling of GDP in cities grouped in neuron no.2 in 2002 and in 2012. (TIF)

S9 Fig Scaling of GDP in cities grouped in neuron no.3 in 2002 and in 2012. (TIF)

S10 Fig Scaling of GDP in cities grouped in neuron no.4 in 2002 and in 2012. (TIF)

S11 Fig Scaling of GDP in cities grouped in neuron no.5 in 2002 and in 2012. (TIF)

S12 Fig Scaling of GDP in cities grouped in neuron no.6 in 2002 and in 2012. (TIF)

S13 Fig Scaling of GDP in cities grouped in neuron no.7 in 2002 and in 2012. (TIF)

S14 Fig Scaling of GDP in cities grouped in neuron no.8 in 2002 and in 2012. (TIF)

S15 Fig Scaling of GDP in cities grouped in neuron no.9 in 2002 and in 2012. (TIF)

S16 Fig Scaling of GDP in cities grouped in neuron no.10 in 2002 and in 2012. (TIF)

S17 Fig Scaling of GDP in cities grouped in neuron no.11 in 2002 and in 2012. (TIF)

S18 Fig Scaling of GDP in cities grouped in neuron no.12 in 2002 and in 2012. (TIF)

S1 File Appendix. **Table A.** Measures of clustering quality _881×*k* = 10_**B** for selected networks of *x* × *y* dimensions. **Table B.** Distribution of the number of cities on the network of 12 neurons. **Table C.** Average values (medians) of the 10 features F1-F10 of cities in every neuron. Real values scaled onto a range [0,1].
(DOCX)
