# Supplementary figures and images for: Scaling Laws in City Growth: Setting Limitations with Self-Organizing Maps
Source: PLoS One. 2016 Dec 22;11(12):e0168753. doi: 10.1371/journal.pone.0168753 (PMC5179107; doi:10.1371/journal.pone.0168753)

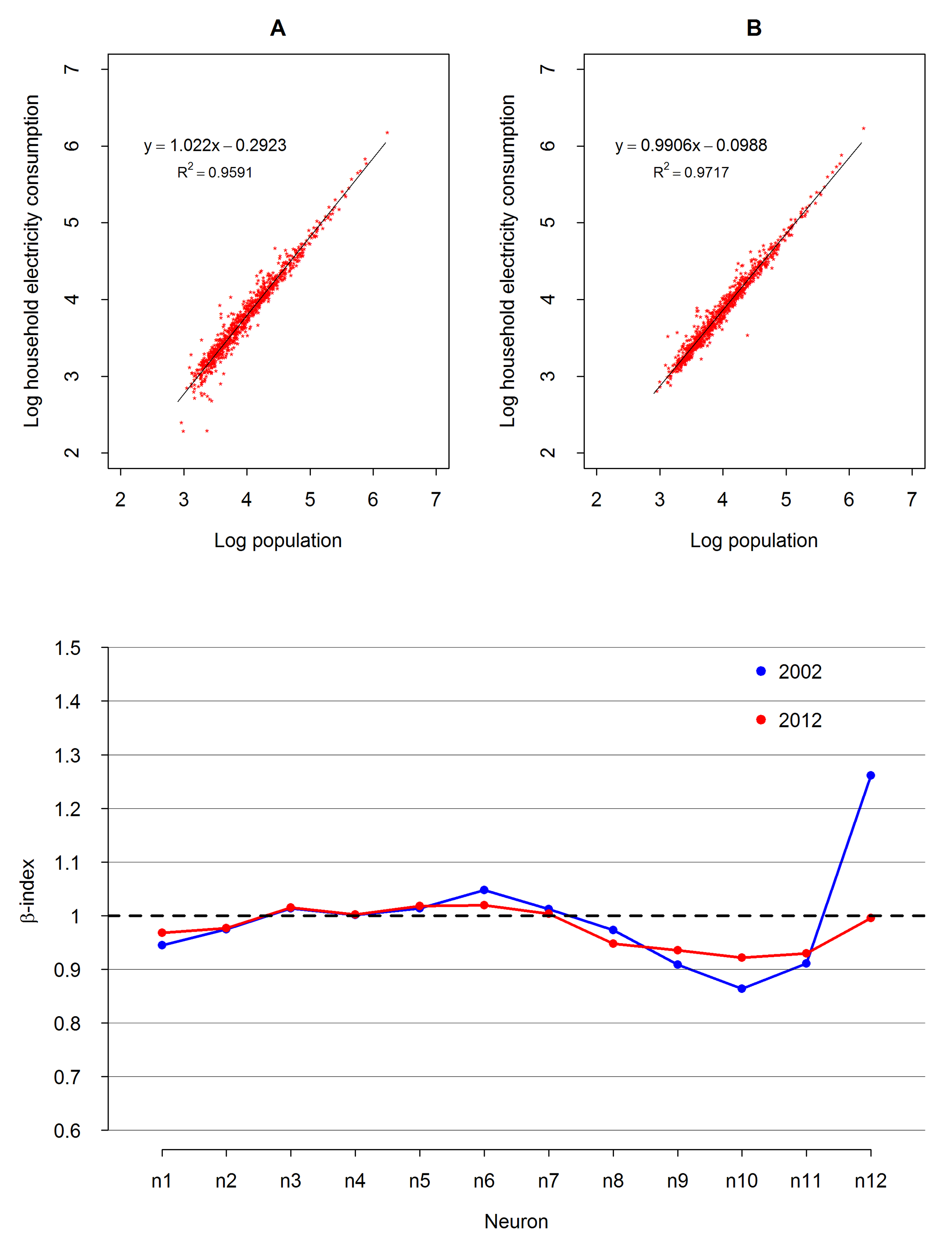

Supplement: S1 Fig — (TIF) [file pone.0168753.s001.tif]

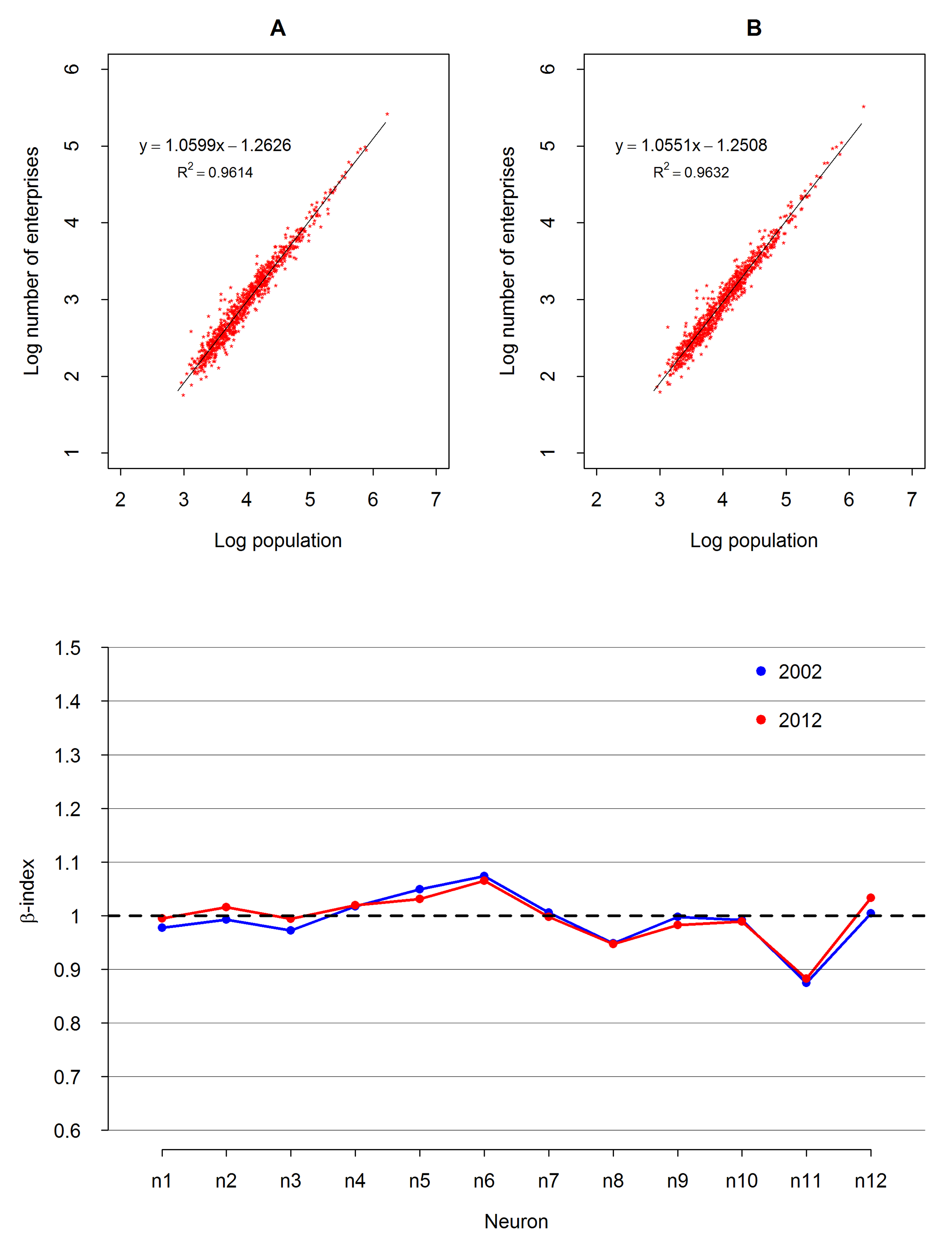

Supplement: S2 Fig — (TIF) [file pone.0168753.s002.tif]

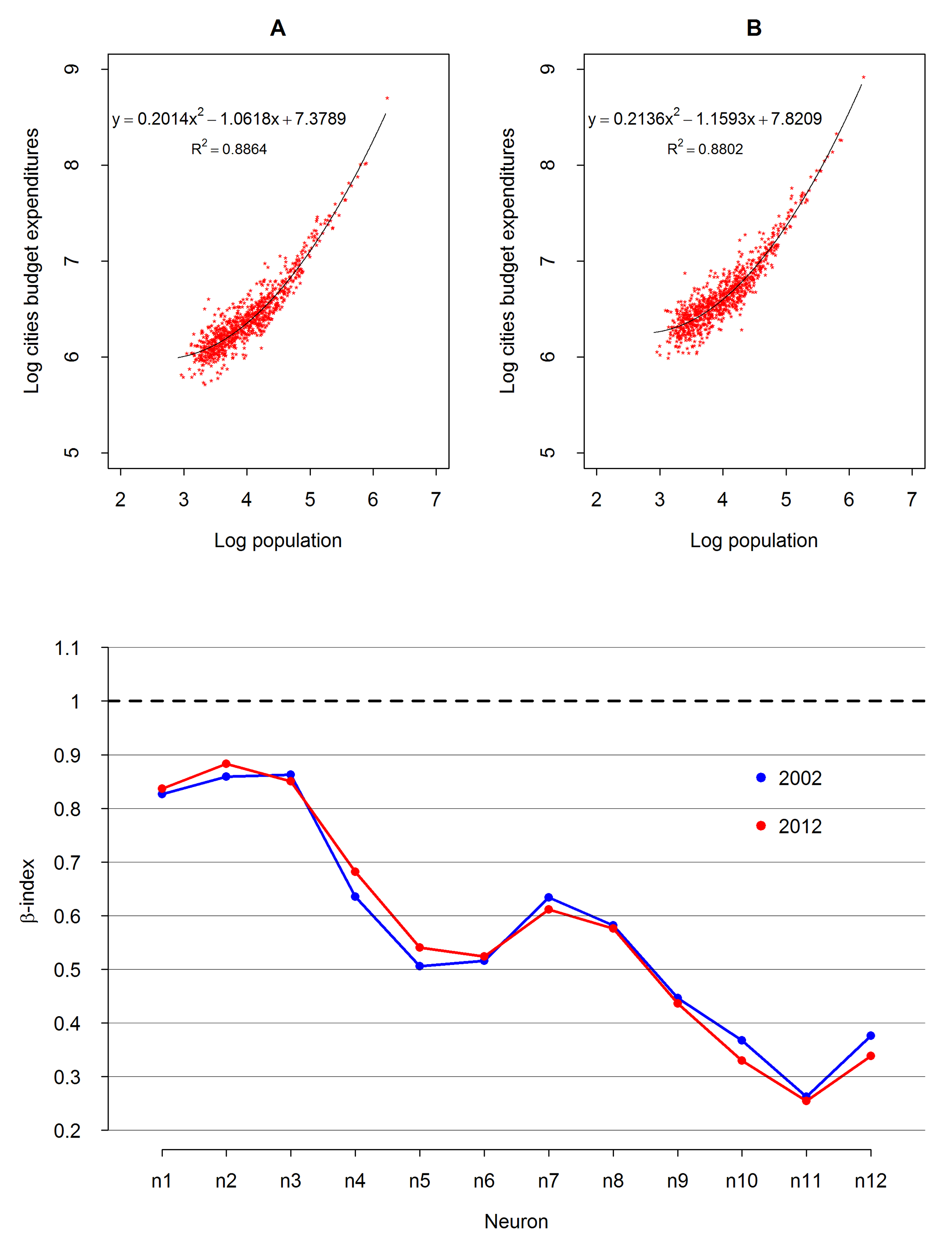

Supplement: S3 Fig — (TIF) [file pone.0168753.s003.tif]

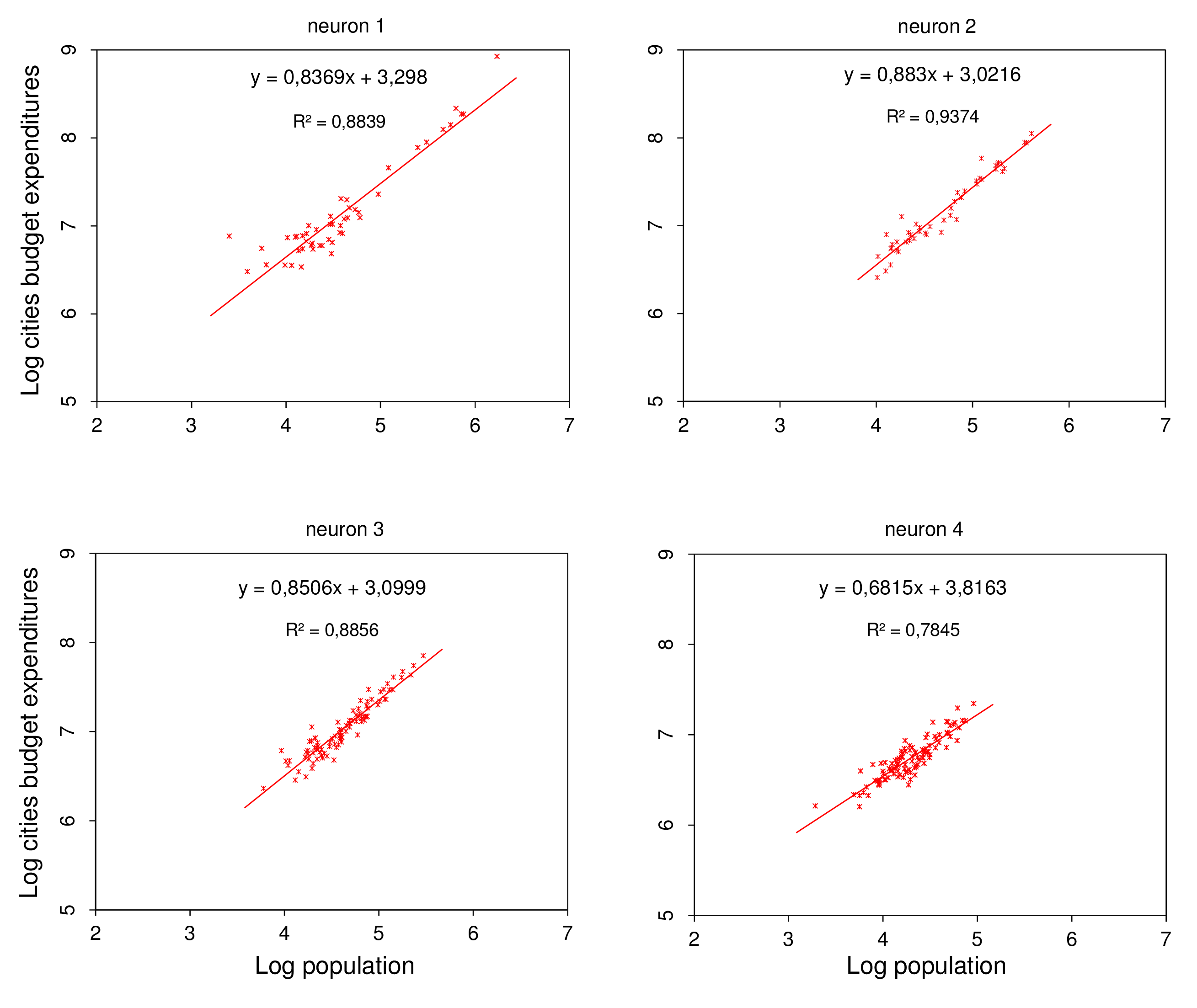

Supplement: S4 Fig — (TIF) [file pone.0168753.s004.tif]

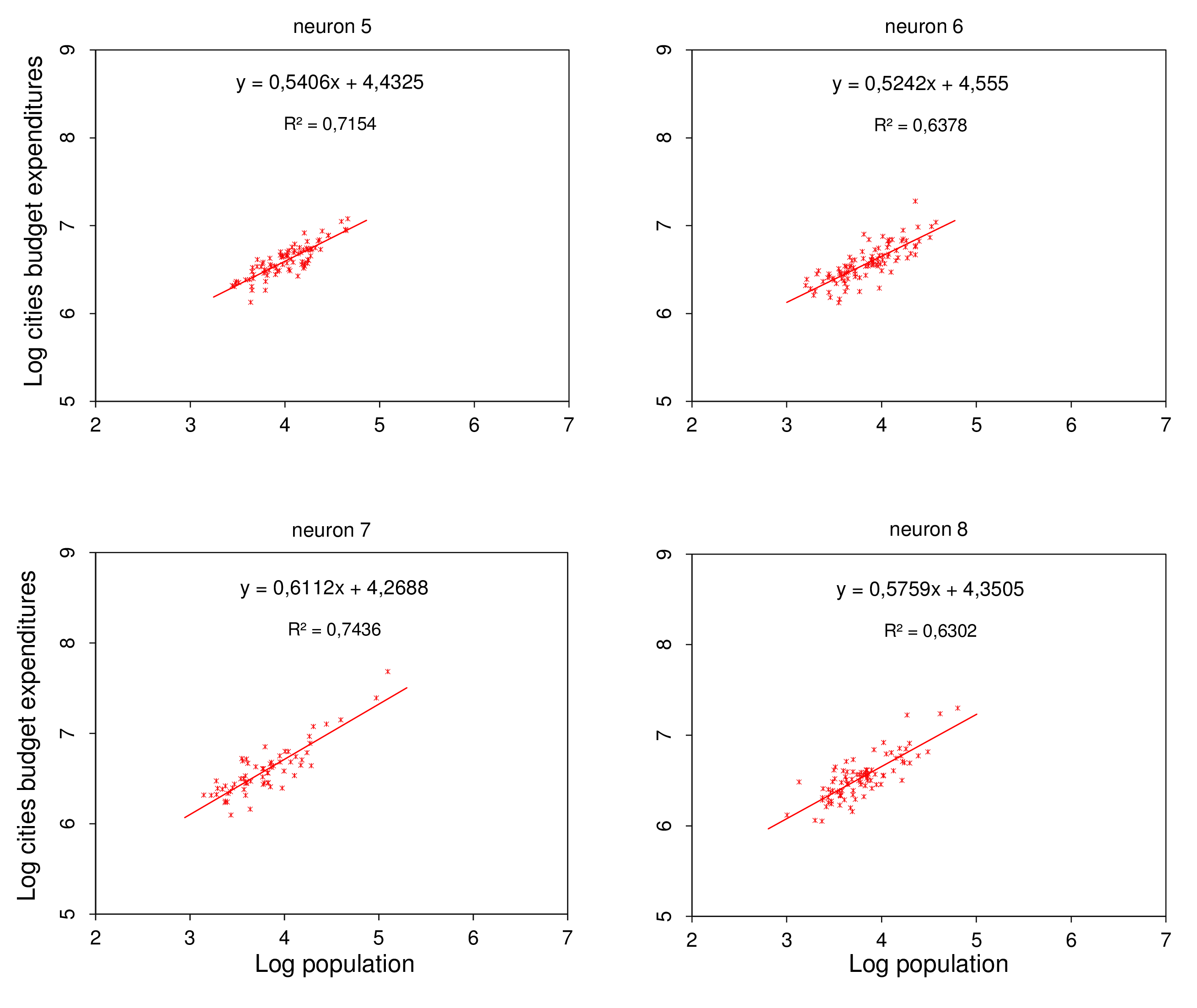

Supplement: S5 Fig — (TIF) [file pone.0168753.s005.tif]

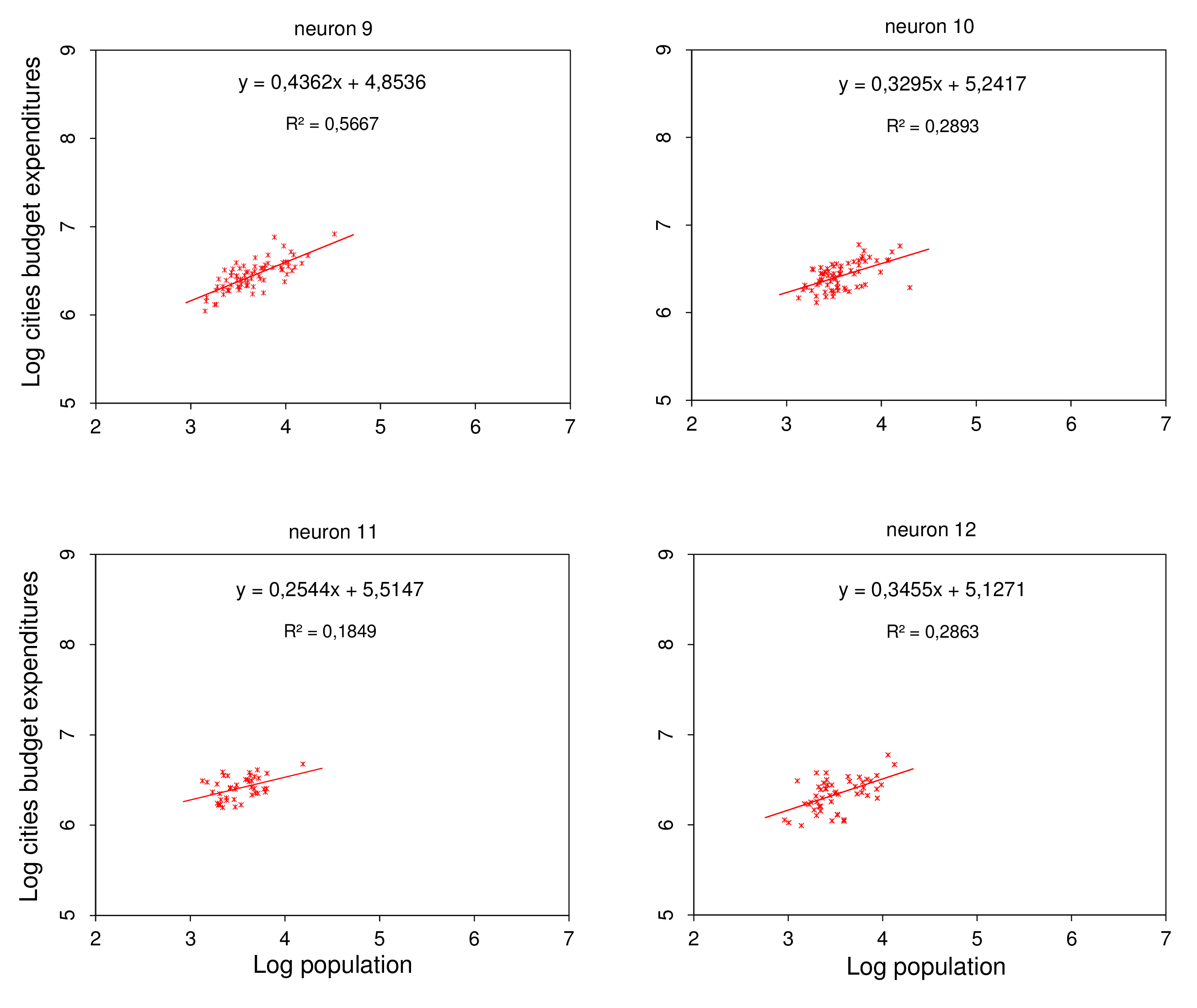

Supplement: S6 Fig — (TIF) [file pone.0168753.s006.tif]

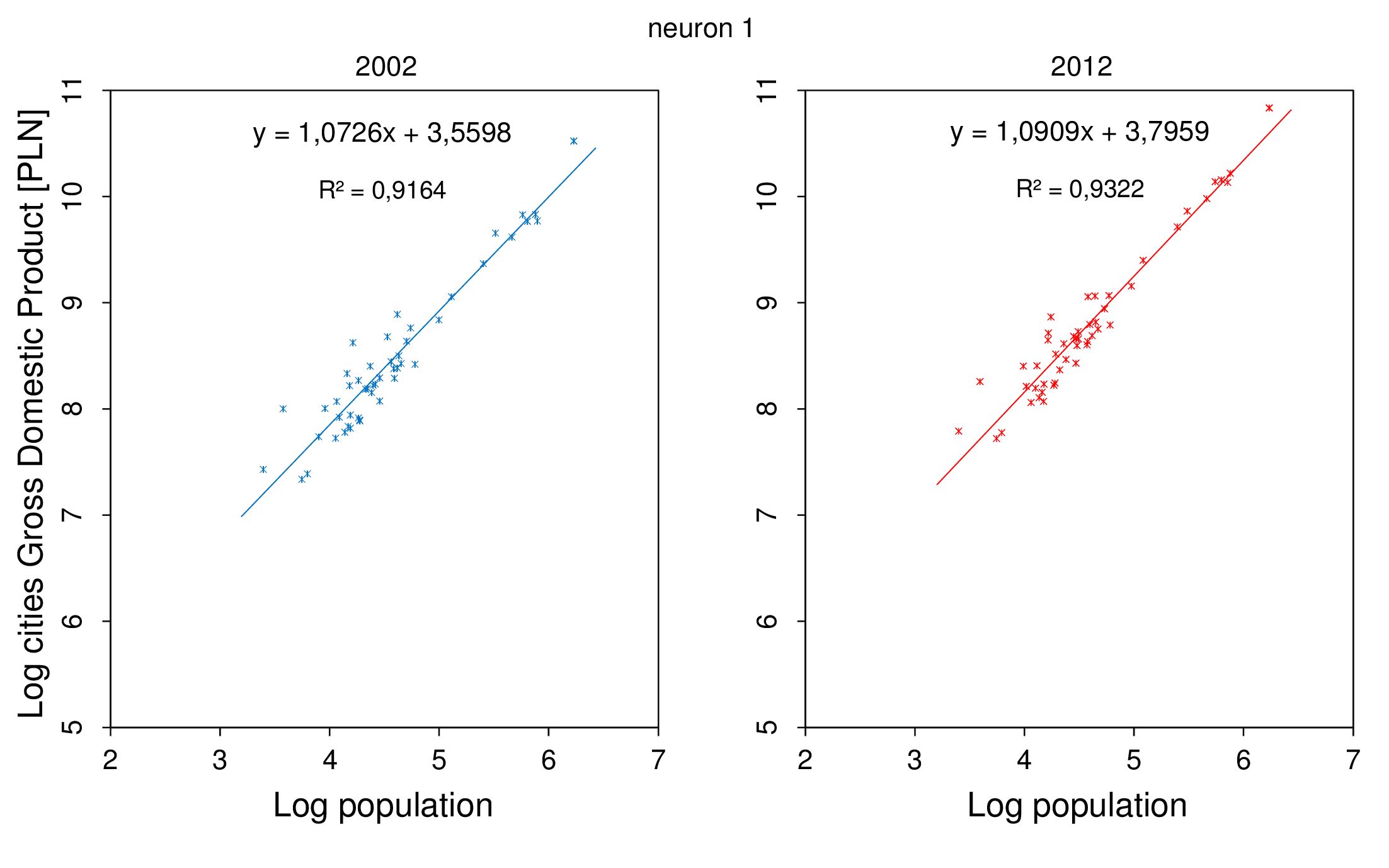

Supplement: S7 Fig — (TIF) [file pone.0168753.s007.tif]

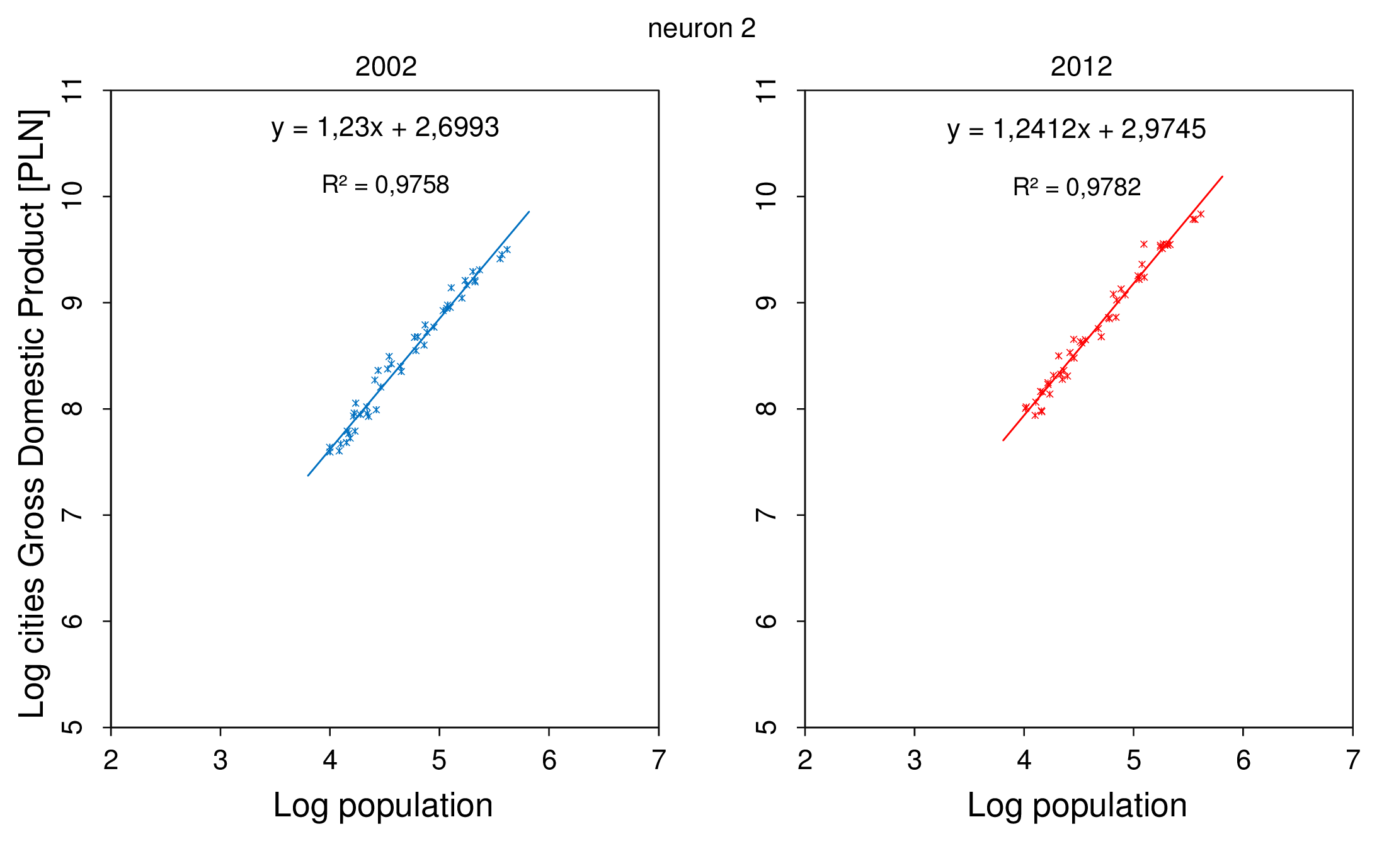

Supplement: S8 Fig — (TIF) [file pone.0168753.s008.tif]

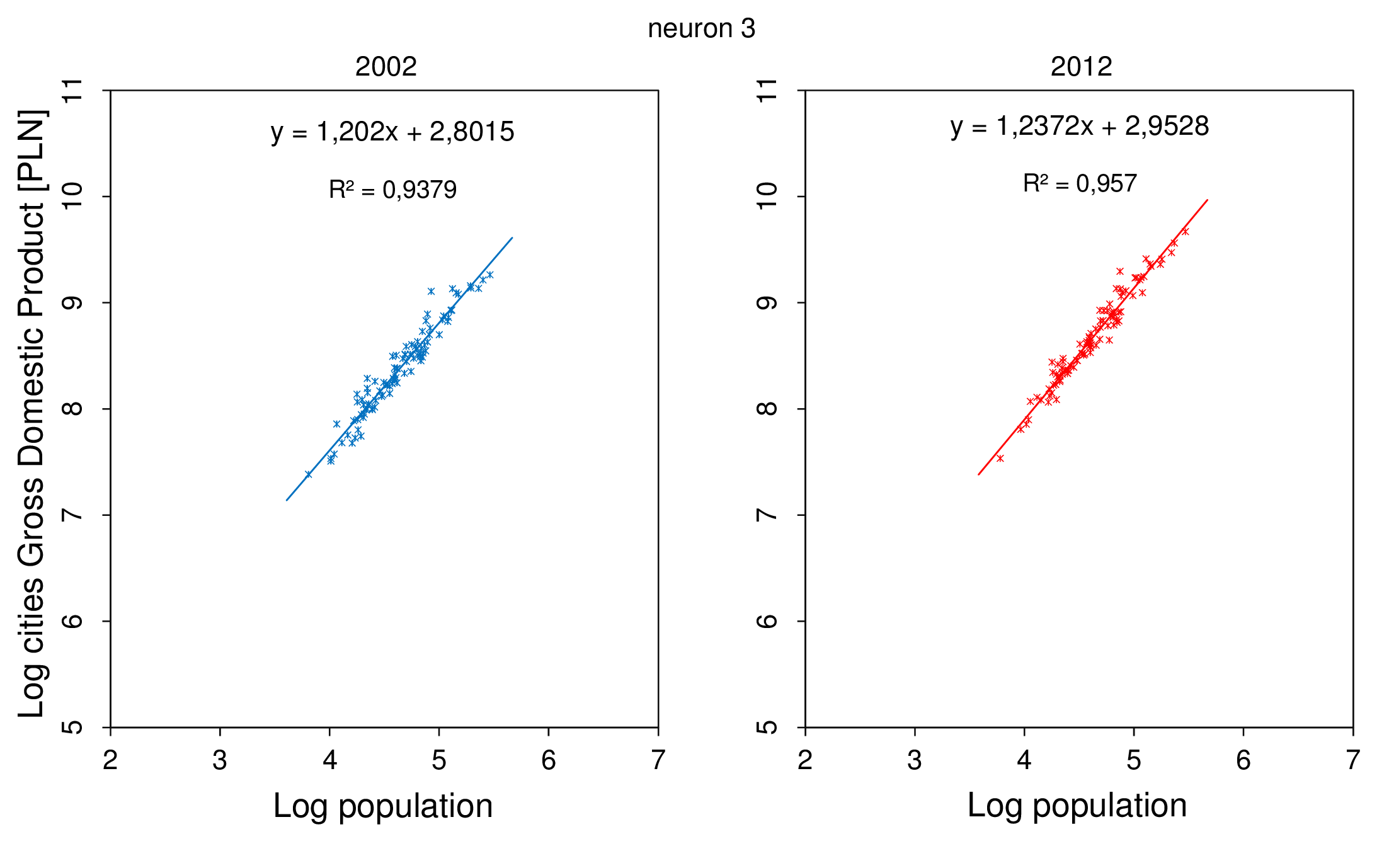

Supplement: S9 Fig — (TIF) [file pone.0168753.s009.tif]

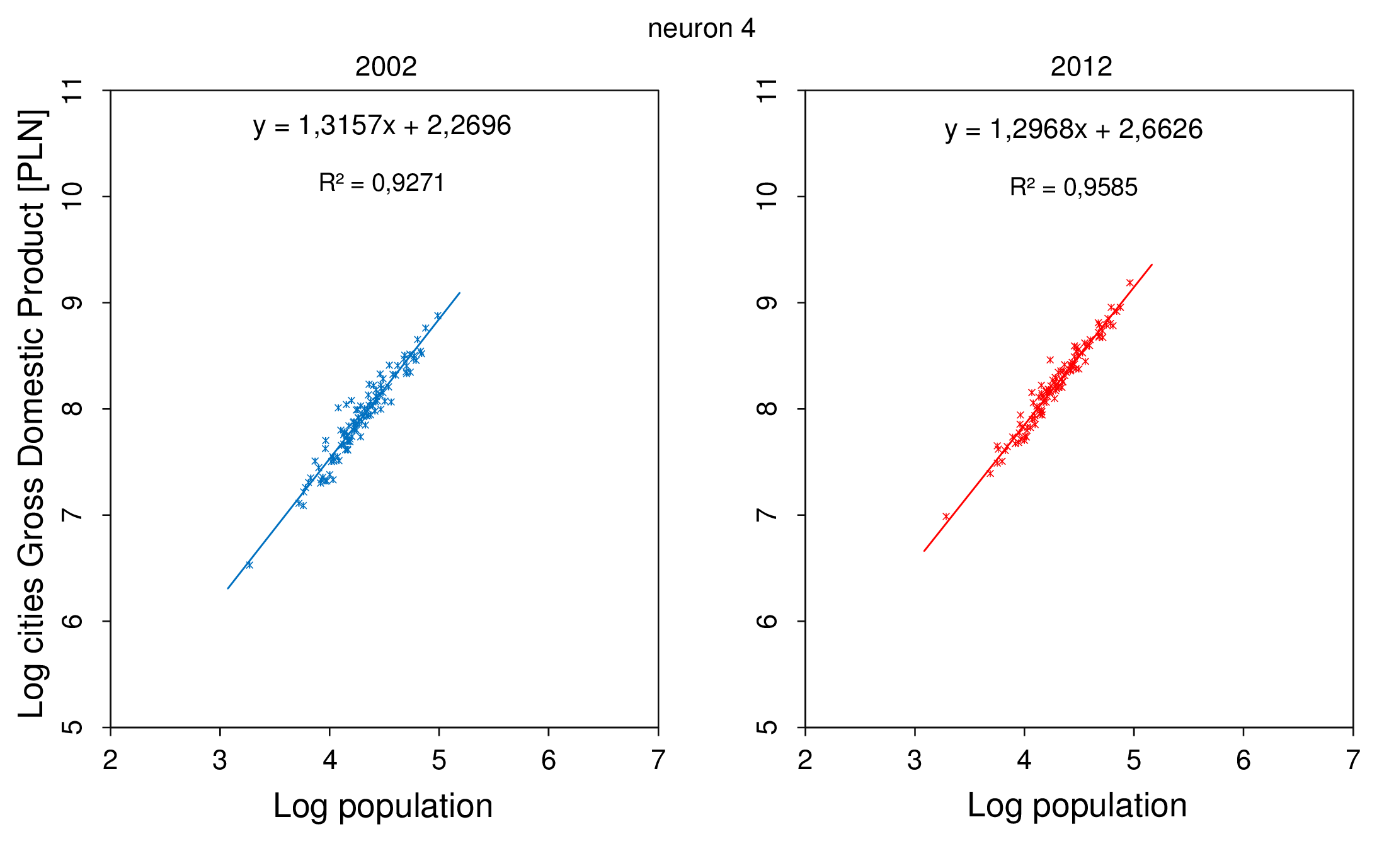

Supplement: S10 Fig — (TIF) [file pone.0168753.s010.tif]

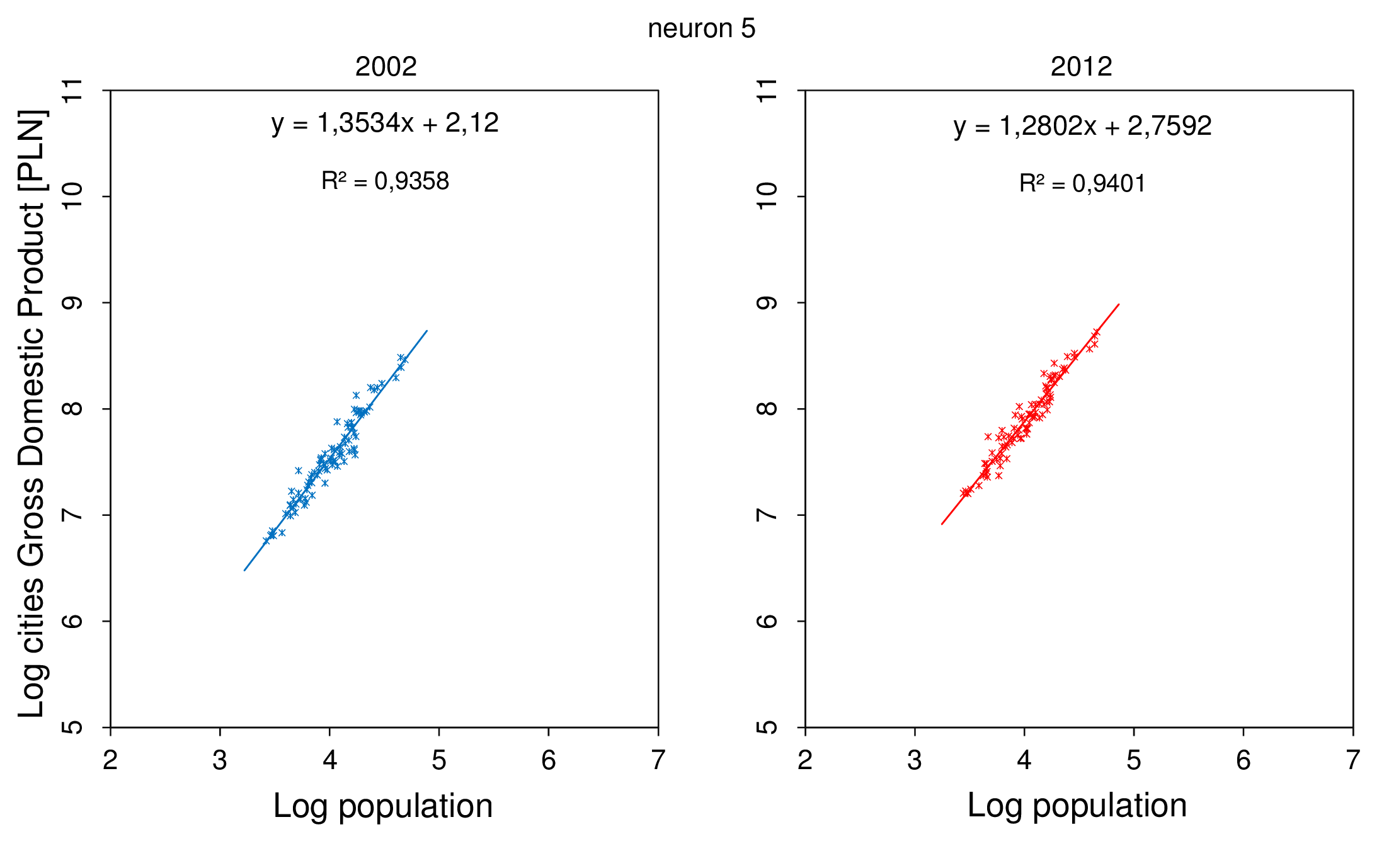

Supplement: S11 Fig — (TIF) [file pone.0168753.s011.tif]

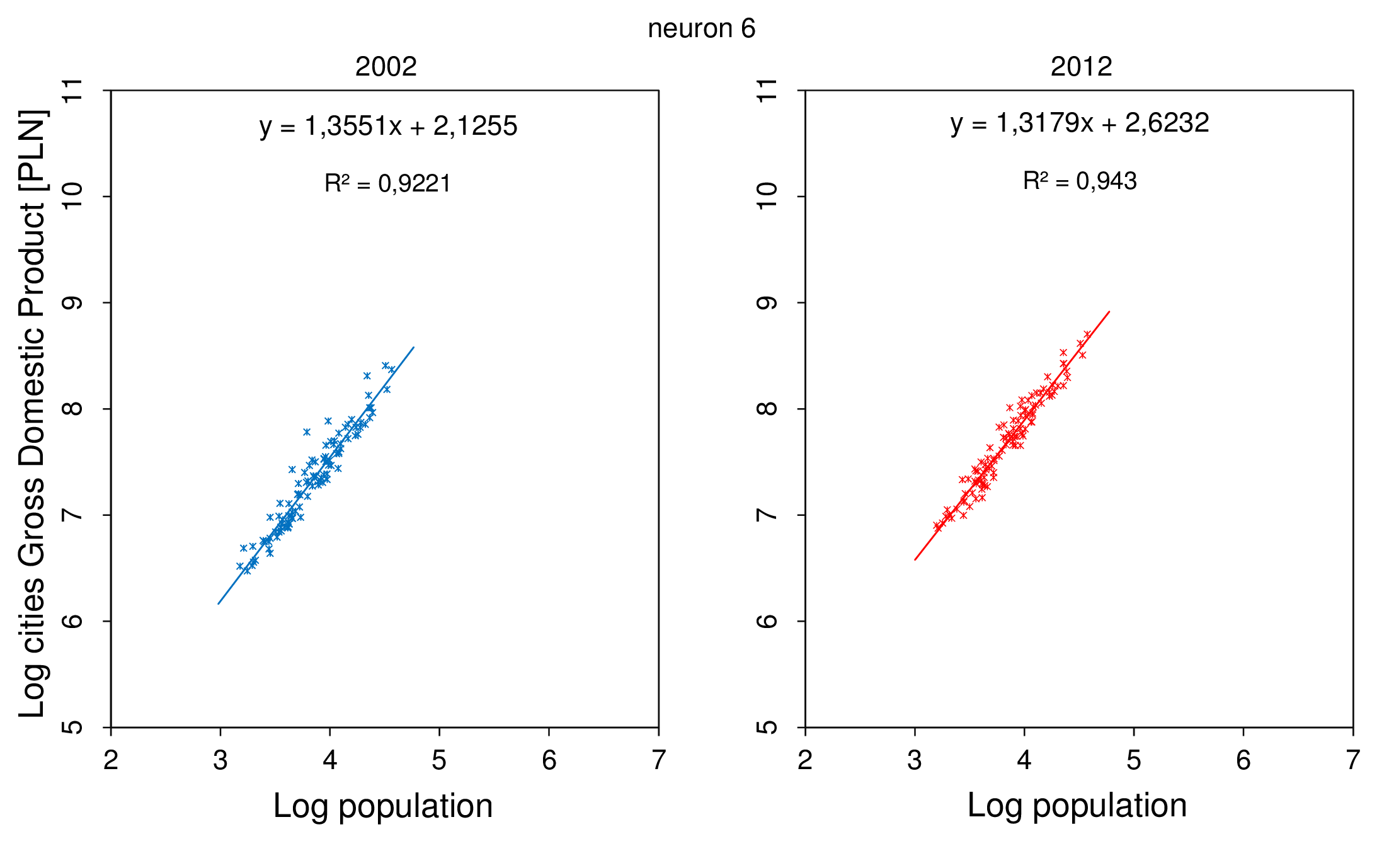

Supplement: S12 Fig — (TIF) [file pone.0168753.s012.tif]

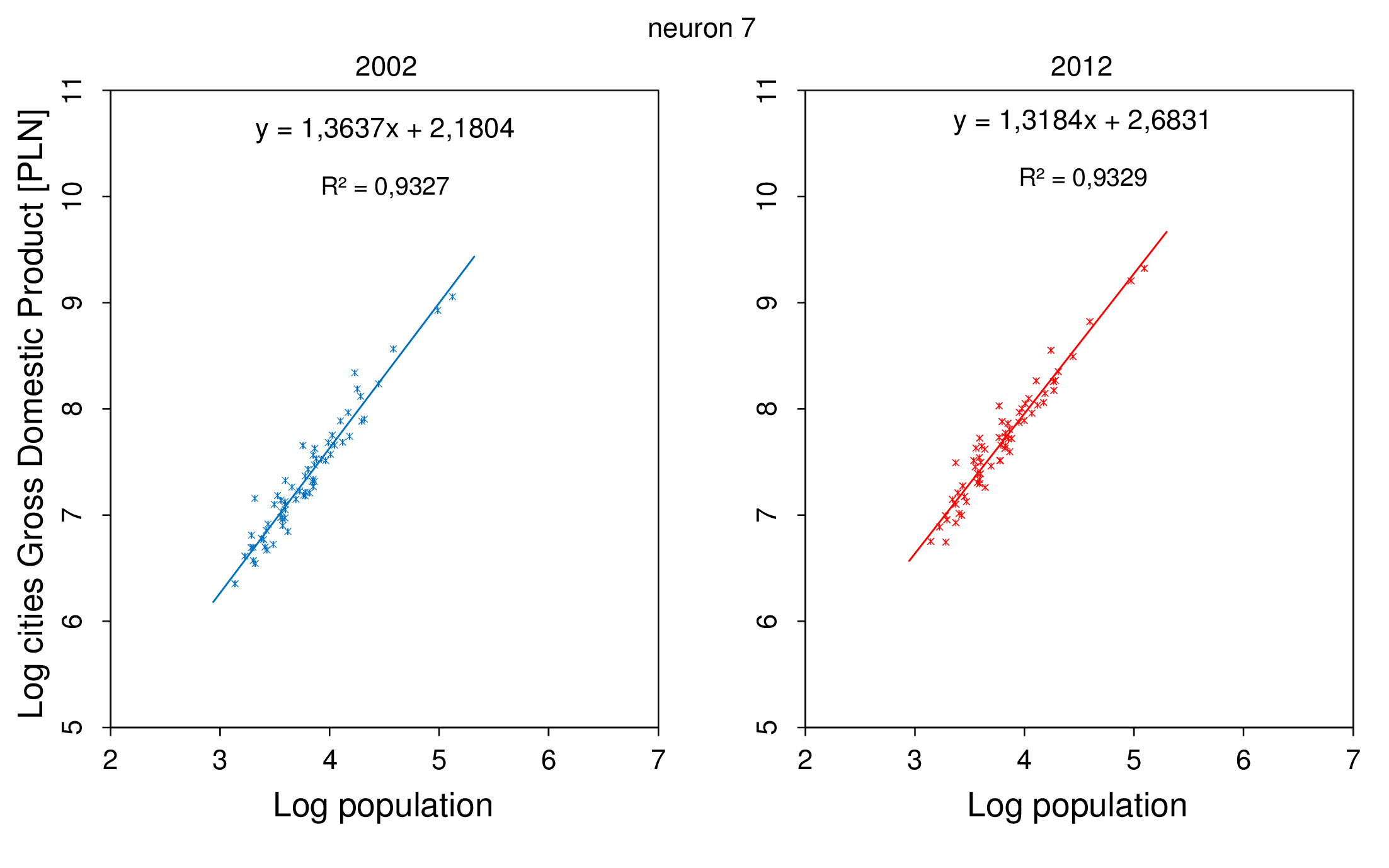

Supplement: S13 Fig — (TIF) [file pone.0168753.s013.tif]

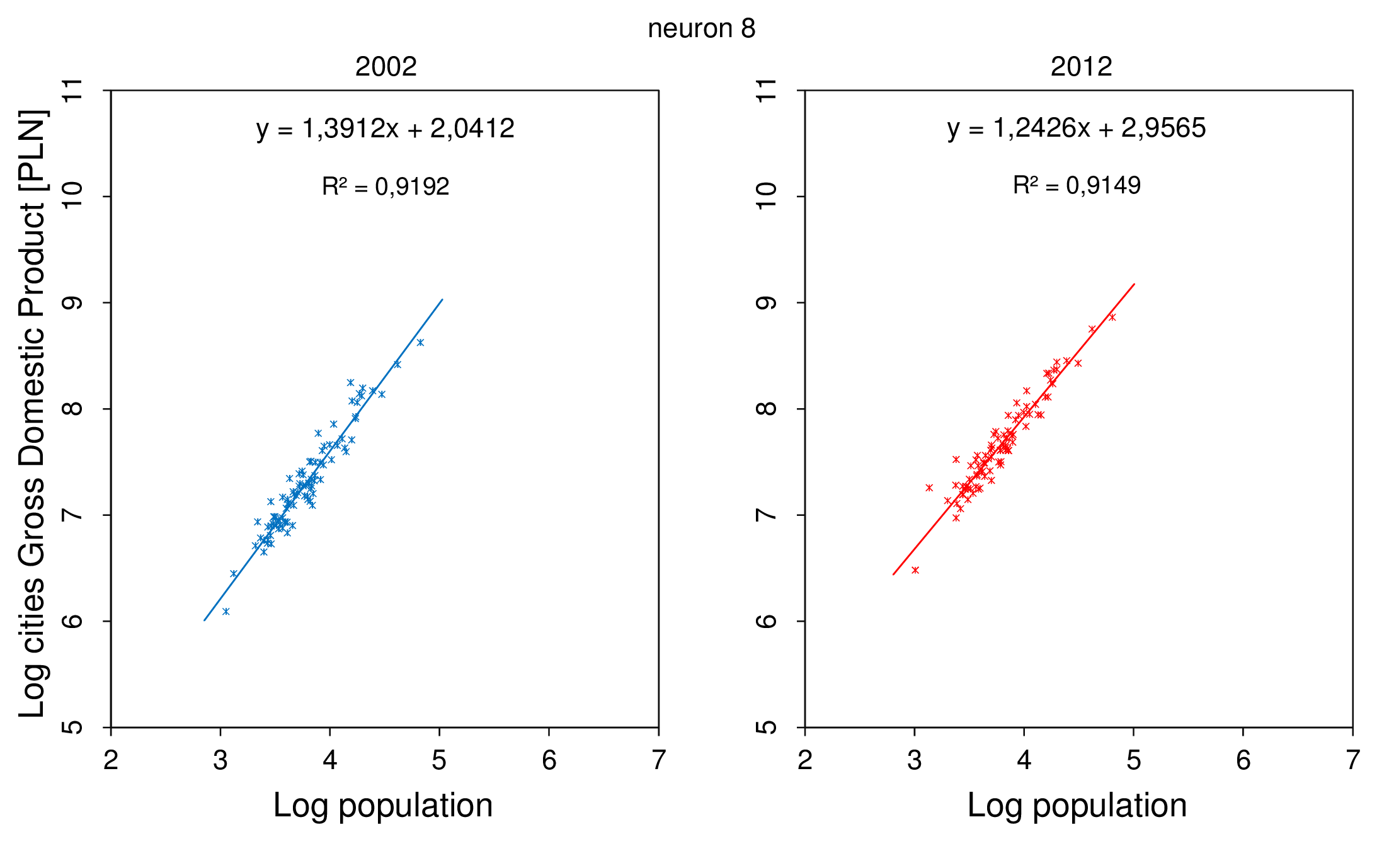

Supplement: S14 Fig — (TIF) [file pone.0168753.s014.tif]

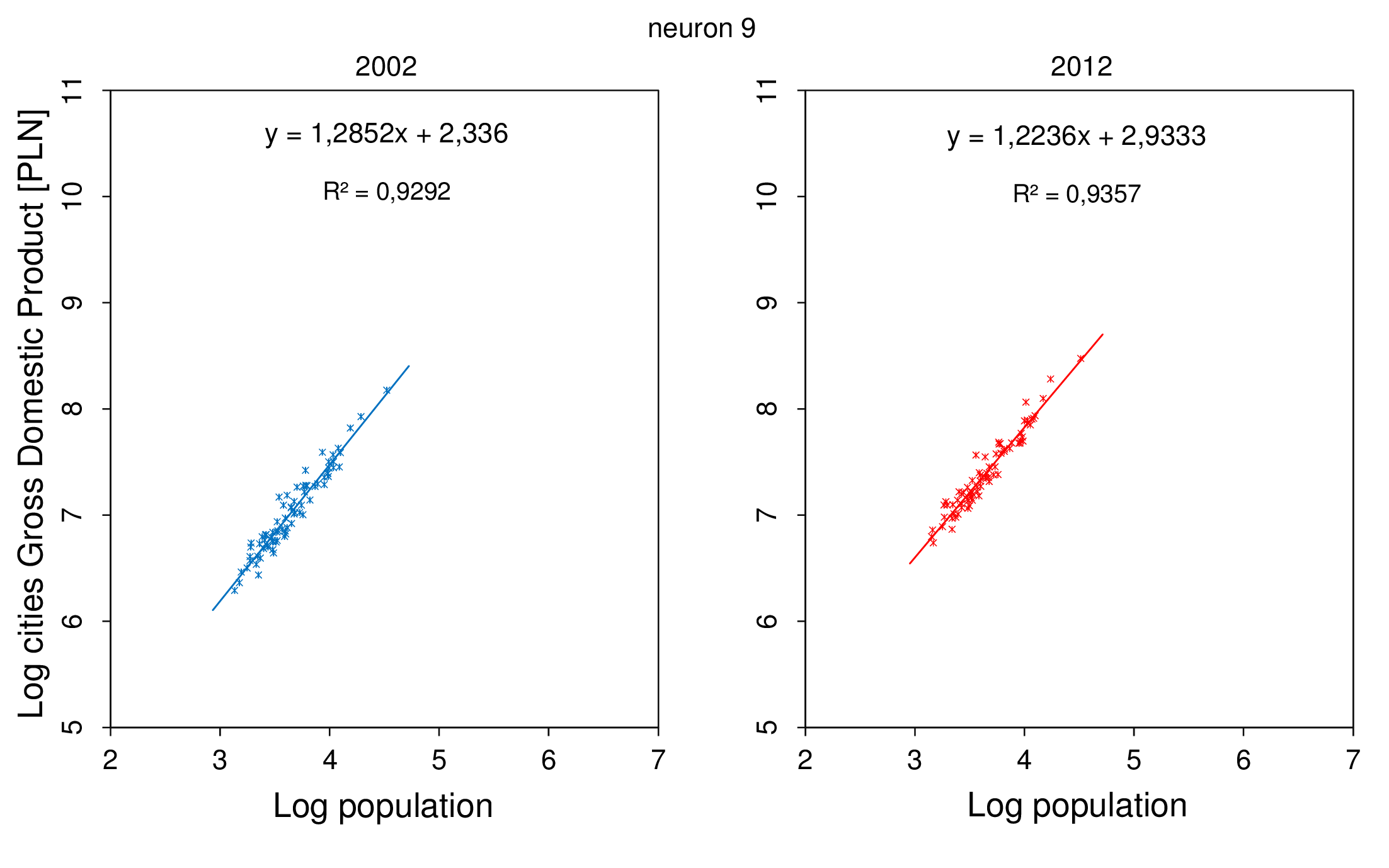

Supplement: S15 Fig — (TIF) [file pone.0168753.s015.tif]

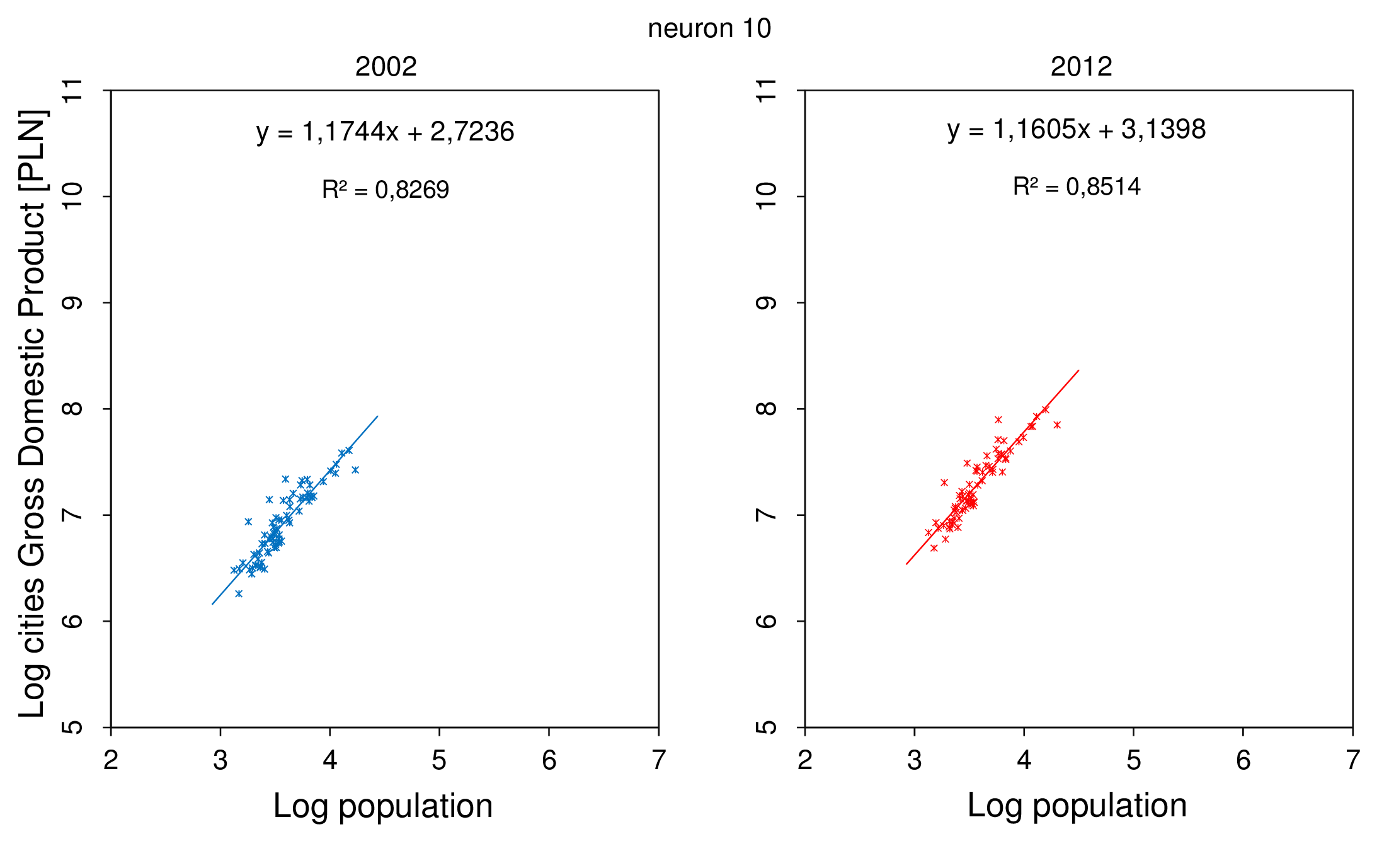

Supplement: S16 Fig — (TIF) [file pone.0168753.s016.tif]

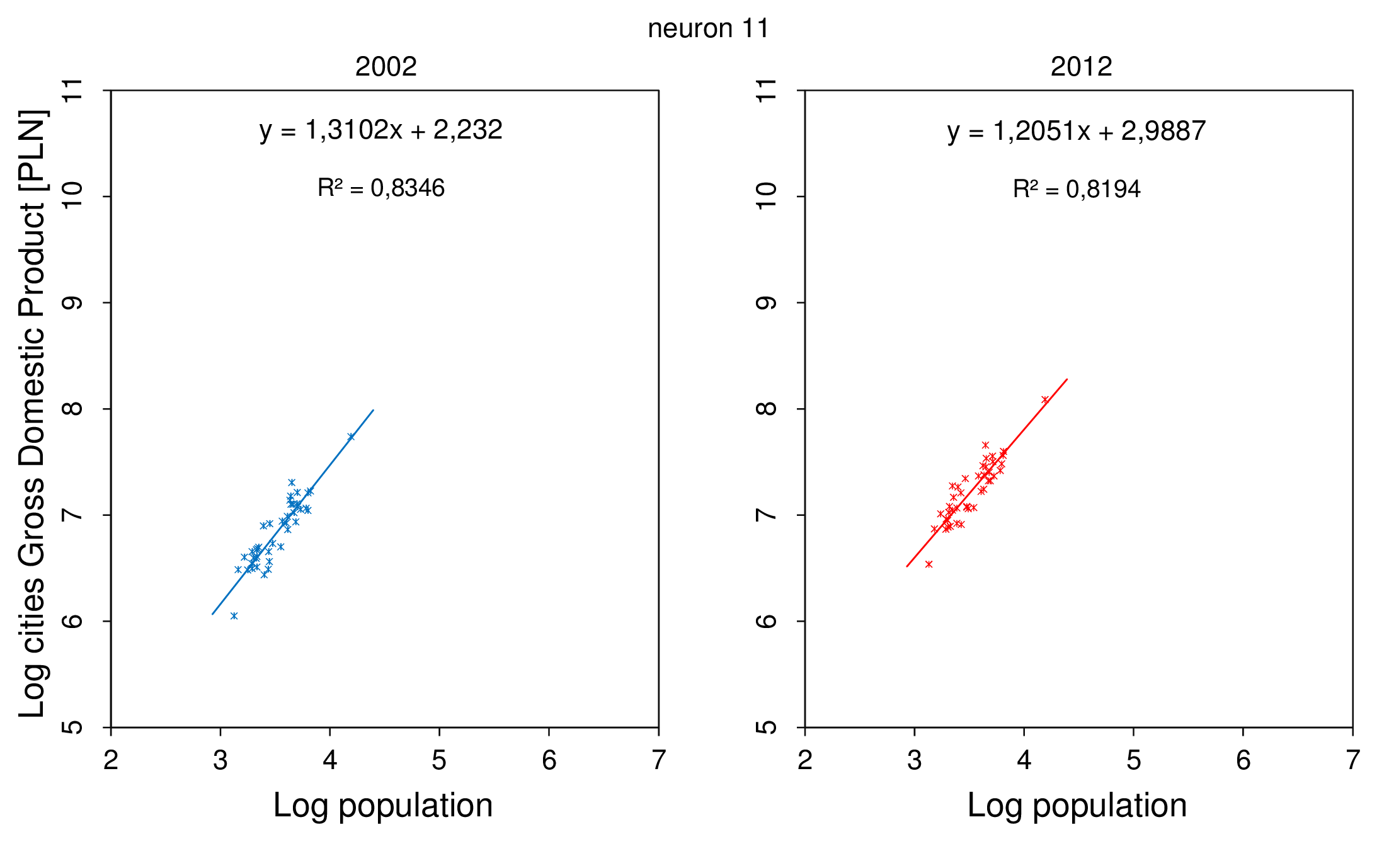

Supplement: S17 Fig — (TIF) [file pone.0168753.s017.tif]

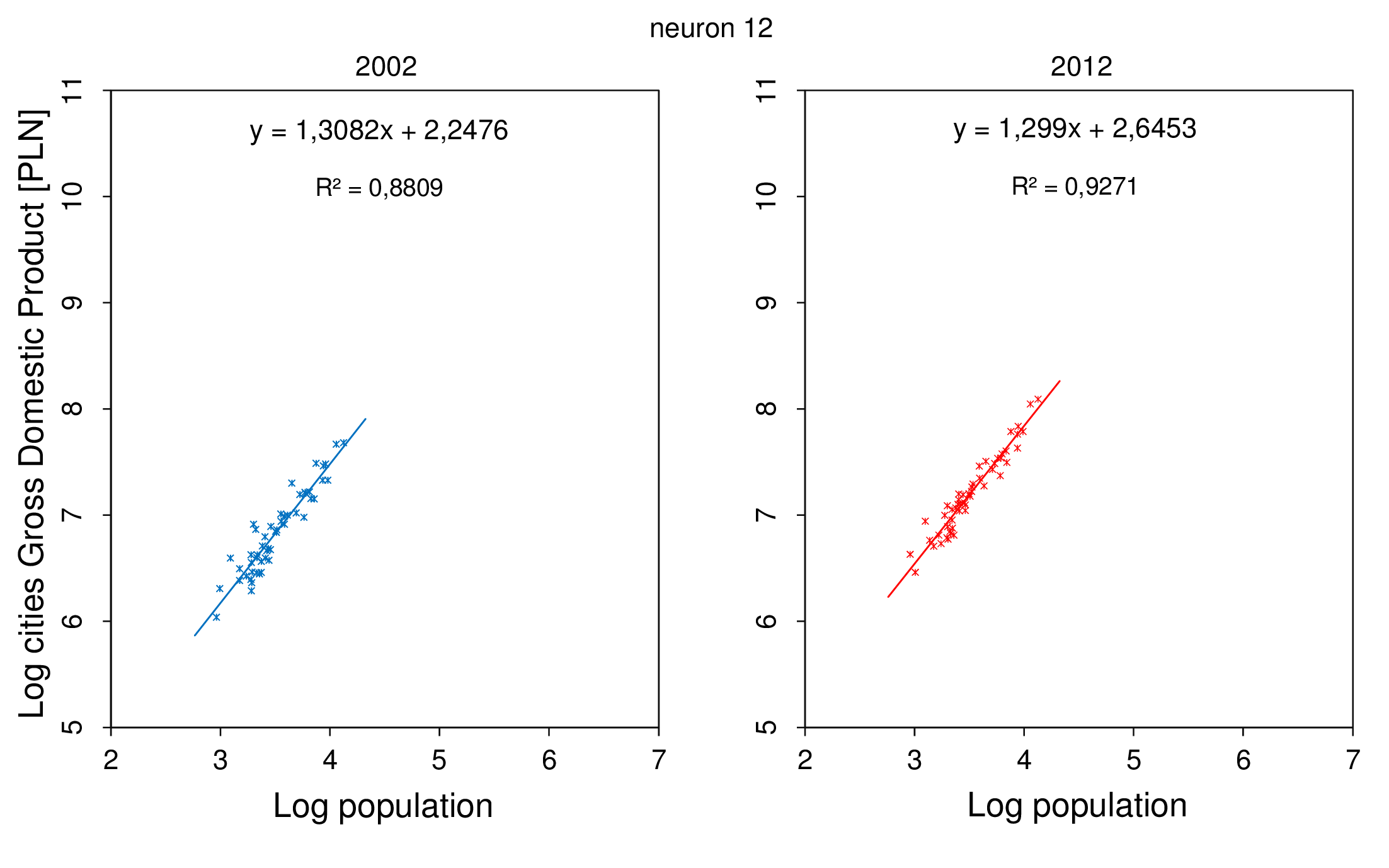

Supplement: S18 Fig — (TIF) [file pone.0168753.s018.tif]
