# Supplementary material for: Scaling Laws in City Growth: Setting Limitations with Self-Organizing Maps
Source: PLoS One. 2016 Dec 22;11(12):e0168753. doi: 10.1371/journal.pone.0168753 (PMC5179107; doi:10.1371/journal.pone.0168753)
Supplement: S1 File — Table A. Measures of clustering quality 881×k = 10B for selected networks of x × y dimensions. Table B. Distribution of the number of cities on the network of 12 neurons. Table C. Average values (medians) of the 10 features F1-F10 of cities in every neuron. Real values scaled onto a range [0,1]. (DOCX) [file pone.0168753.s019.docx]

# Appendix

S1-S18 Figs

Tables A-C

References [22-31]

# Materials and Methods.

**Data sources.**

The data were acquired between August 2013 and October 2014 from TERYT and NTS databases in the freely accessible Local Data Bank of the Central Statistical Office (http://www.stat.gov.pl/gus [accessed: 20.08.2013 - 16.04.2014].)

**Indicators describing cities.**

We selected the features on the basis of which cities were grouped on the assumption that there is at least a qualitative analogy between urban development and the evolution of ecosystems (both the city and the ecosystems are complex systems, dynamic and open in the sense of the flow of matter and energy). Moreover, due to self-organization, cities remain in a state far from equilibrium.
This in turn directed our attention to the issues of energy flows in a given city. It seems that the description of metabolism should also include description of the characteristics relevant to the growth and development of the city.
In the science of ecosystems, the issues related to energy flows are indeed widely studied.
Existing findings can be summarized by naming functions describing the flow of matter and energy, whose optimum value seems to be achieved by stabilized ecosystems. This is the size of exergy [22], the size of emergy and transformity [23], systems power and its indirect effects [24] , and finally ascendency as a function of an ecosystem’s trophic network, which depicts the system’s ability to develop (evolution, succession) despite interfering changes in the environment.
On the basis of “tracking” the size of energy flows between the individual elements (nodes) of the network, one can describe the complexity of the system and the amount of energy dissipated [25-27]. According to the latter, ascendency expresses the boundaries of each ecosystem’s growth and development [28]. Threats to the system are deferred by increasing the number and diversity of system components [29].

This similarity to ecosystems could explain the accelerating growth of cities [30]. By increasing their population, area, and a diversity of links, cities increase the quantity and variety of the system elements, while at the same time increasing the speed and efficiency of the acquisition, processing, and – ultimately – dissipation of energy [22, 24]. This is nothing more than the long-researched area of metabolism [15].

Therefore, the chosen features are expected to describe this property in the cities observed.

The indicators (features) and the basis of their creation are discussed below. The numbering is consistent with that used in the analysis of the results of clustering by means of SOM.

Feature 1 (F1): [Factorized area of forests / city area]
The factorized area of forest land in total (the factor limits the amount of absorbed solar energy on the basis of growing season time span, climate zone, and the value of annual insolation). The adjusted value is based on the area of the city.
Feature 1. describes the amount of biomass energy possible to harness and store in the forests within the city limits.

Feature 2 (F2): [total usable floor space of (all) dwellings / city area]

This feature describes the energy embodied in the structure of the buildings. Consistent data were attainable only for residential buildings.

Feature 3 (F3): [total income of city residents (in terms of energy intensity) / city area]

One can consider this feature in a twofold sense: as a description of city income, or as description of total energy consumption in respect to its surface. It is assumed that: a) the gross product can be determined from the revenue side, b) one can calculate the total energy consumption required to produce one unit of gross domestic product upon the energy intensity of the global economy of the country. Capital flows and energy flows are considered to be equivalent [31].
The quantity of the energy consumed is corrected using the deviation of the average power consumption in the city, taken from the average consumption of electricity in Poland. The purpose of the adjustment is to implement the diversity of energy consumption dependent upon the local structure of the economy, transport, or climate.
Features associated with the economic sector (both this and the following) are based on the assumption that the income of the residents of a given city is the same as the income of the residents throughout the entire municipality. With regard to small cities (towns that have not achieved county rights), it is a necessary simplification due to the lack of the separation of accounting and reporting to the CSO. It is expected that, in fact, the income of urban residents is higher than the average income throughout the municipality.

Feature 4 (F4): [budget expenditures of the local government / total income of city residents]

This feature determines the proportion of budget expenditures necessary to maintain the operation of the city to the income of its inhabitants. Tasks, as financed by the municipal budget, concern anything that meets the collective needs of the community, not reserved by law for other entities. These include, for example, public health, education, culture, local public transport, the supply of energy and water, disposal and waste water treatment, public order, and the maintenance of municipal buildings and public facilities. From the point of view of energy, one can say that it shows the energy efficiency of the city.

Feature 5 (F5): [budget expenditures per capita]

Like the previous feature, (F5) determines the proportion of expenditures necessary to keep the city functioning, but in per capita terms.

Feature 6 (F6): [number of enterprises / city area].

This feature describes the multiplicity of the forms of energy conversion and was calculated based on the number of registered economic activities.

Feature 7 (F7): [number of enterprises per capita]

This feature determines the number of forms of energy conversion - in relation to the number of inhabitants.

Feature 8 (F8): [Enterprise Diversity Index (EDI)]

[$EDI=1-\Sigma\left( \frac{number of enterprises in a category (by PKD)}{total number of enterprises} \right)$^2^ ] (1)

This feature shows the variety of economic activities, in this respect also the variety of energy conversion forms.

Feature 9 (F9): [gas consumption in households / total usable floor space of dwellings]

Assuming that gas consumption for living purposes is negligibly small compared to its consumption for heating and is constant across all cities, this feature describes the technical condition of the buildings (degree of energy dissipation in the heat).

Feature 10 (F10): [electricity consumption in households / total usable floor space of dwellings]

This feature determines the consumption of highly-processed forms of energy. In contrast to the (F9) technical condition of the buildings, this has a relatively small impact on its value.

# ****Results of grouping the cities.****

The following are the most important results of the clustering.

The quality of clustering is shown in Table A, across several networks of selected configurations. One can see, for example, that of the three 12-neuron networks with different configurations (shaded area), the network size $x=1\times y=12$ has the lowest error value $BIC=769.7$, and one of the lowest errors $e=2.003$.

**Table A.** **Measures of clustering quality${}_{\text{881 }\times\text{ }k=10}\mathbf{B}$ for selected networks of $x\times y$dimensions.**

| ***x*** | ***y*** | ***BIC*** | ***e*** | ***f*** [%] | ***x*** | ***y*** | ***BIC*** | ***e*** | ***f*** [%] | ***x*** | ***y*** | ***BIC*** | ***e*** | ***f*** [%] |
| --- | --- | --- | --- | --- | --- | --- | --- | --- | --- | --- | --- | --- | --- | --- |
| 1 | 2 | 911.8 | 2.567 | 0.000 | 1 | 14 | 771.2 | 1.977 | 5.221 | 2 | 12 | 867.2 | 2.043 | 0.454 |
| 1 | 3 | 841.0 | 2.336 | 0.000 | 1 | 15 | 770.5 | 1.964 | 9.421 | 3 | 3 | 907.5 | 2.406 | 0.000 |
| 1 | 4 | 813.3 | 2.256 | 0.000 | 2 | 2 | 929.4 | 2.587 | 0.000 | 3 | 4 | 899.2 | 2.325 | 0.114 |
| 1 | 5 | 803.1 | 2.210 | 0.000 | 2 | 3 | 886.1 | 2.412 | 0.000 | 3 | 5 | 891.2 | 2.249 | 0.000 |
| 1 | 6 | 792.9 | 2.174 | 0.454 | 2 | 4 | 857.1 | 2.278 | 0.000 | 3 | 6 | 892.0 | 2.195 | 0.000 |
| 1 | 7 | 786.7 | 2.140 | 0.227 | 2 | 5 | 846.3 | 2.222 | 0.000 | 3 | 7 | 898.6 | 2.159 | 0.000 |
| 1 | 8 | 778.3 | 2.092 | 1.476 | 2 | 6 | 844.1 | 2.191 | 0.000 | 4 | 4 | 909.9 | 2.300 | 0.000 |
| 1 | 9 | 773.0 | 2.060 | 2.951 | 2 | 7 | 846.3 | 2.156 | 0.114 | 4 | 5 | 908.2 | 2.195 | 0.000 |
| 1 | 10 | 773.6 | 2.032 | 2.724 | 2 | 8 | 849.6 | 2.142 | 0.000 | 4 | 6 | 914.0 | 2.142 | 0.000 |
| 1 | 11 | 772.7 | 2.022 | 2.724 | 2 | 9 | 854.1 | 2.117 | 0.114 | 5 | 5 | 929.6 | 2.183 | 0.000 |
| **1** | **12** | **769.7** | **2.003** | **3.178** | 2 | 10 | 857.7 | 2.089 | 0.341 | 5 | 6 | 940.6 | 2.120 | 0.000 |
| 1 | 13 | 775.3 | 2.005 | 5.789 | 2 | 11 | 862.0 | 2.057 | 0.795 | 6 | 6 | 971.6 | 2.097 | 0.000 |

We are not quite sure why cities finally grouped in linear manner, even though features describing cities seem to have many interdependencies.

Moreover, the fact that the best map happened to be linear might lead to noticing similarities with ranking-grouping methods. But this would be misleading: applying any ranking-grouping method would be an a priori assumption on weighs of features describing cities. The analyzed final map is a result of grouping – not an established topology. It is the best statistical model of analyzed data, and it also allows further research on interdependencies and weighs of features describing cities.

Each of the 12 neurons of the SOM network (Table B) is comprised of cities that are similar to each other with respect to all analyzed features, and the differences between cities are smaller as the proximity of the neurons to which they were assigned gets closer.

**Table B.** **Distribution of the number of cities on the network of 12 neurons.**

| Neuron | 1 | 2 | 3 | 4 | 5 | 6 | 7 | 8 | 9 | 10 | 11 | 12 | Total |
| --- | --- | --- | --- | --- | --- | --- | --- | --- | --- | --- | --- | --- | --- |
| Number of cities | 49 | 47 | 96 | 107 | 87 | 101 | 65 | 89 | 75 | 71 | 42 | 52 | 881 |
| % | 5.6 | 5.3 | 10.9 | 12.1 | 9.9 | 11.5 | 7.4 | 10.1 | 8.5 | 8.1 | 4.8 | 5.9 | 100% |

The values of the features are difficult to compare with each other due to the fact that they are expressed as different units. Therefore, Table C contains the median characteristics, both real and scaled on a range $\left[0,1\right]$ , so that if a given neuron had the smallest value of a given feature, the scaled value will be zero. Similarly, the largest of the 12 values of the features will be 1. This shows the relationships between the average values of the features in the neurons that are to be examined in more detail in ongoing studies.

**Table C.** **Average values (medians) of the 10 features F1-F10 of cities in every neuron. Real values scaled onto a range [0,1].**

| Neuron | Scale | F1 | F2 | F3 | F4 | F5 | F6 | F7 | F8 | F9 | F10 |
| --- | --- | --- | --- | --- | --- | --- | --- | --- | --- | --- | --- |
| 1 | original | 0.0576 | 744953 | 414899 | 0.0189 | 366.1 | 2.738 | 0.133 | 0.8322 | 89.86 | 34.96 |
|  | [0,1] | 0.177 | 1 | 1 | 0 | 0.663 | 1 | 1 | 1 | 0.438 | 1 |
| 2 | orig. | 0.0667 | 556791 | 245917 | 0.0212 | 292.2 | 2.0075 | 0.1193 | 0.8311 | 65.84 | 31.97 |
|  | [0,1] | 0.221 | 0.73 | 0.587 | 0.016 | 0.296 | 0.722 | 0.765 | 0.922 | 0.122 | 0.731 |
| 3 | orig. | 0.0544 | 443731 | 154199 | 0.0213 | 240 | 1.5438 | 0.1061 | 0.8289 | 56.6 | 29.46 |
|  | [0,1] | 0.162 | 0.568 | 0.363 | 0.016 | 0.037 | 0.545 | 0.539 | 0.759 | 0 | 0.506 |
| 4 | orig. | 0.0576 | 372733 | 109651 | 0.0358 | 232.6 | 1.2694 | 0.1005 | 0.8267 | 65.05 | 28.81 |
|  | [0,1] | 0.177 | 0.466 | 0.254 | 0.116 | 0 | 0.441 | 0.443 | 0.602 | 0.111 | 0.447 |
| 5 | orig. | 0.0384 | 321031 | 86722 | 0.058 | 259.8 | 1.1048 | 0.1015 | 0.8235 | 98.04 | 30.19 |
|  | [0,1] | 0.084 | 0.392 | 0.198 | 0.269 | 0.135 | 0.378 | 0.461 | 0.372 | 0.545 | 0.572 |
| 6 | orig. | 0.0212 | 258144 | 68442 | 0.0834 | 346.9 | 0.9106 | 0.1055 | 0.8202 | 132.58 | 32.34 |
|  | [0,1] | 0 | 0.302 | 0.153 | 0.443 | 0.568 | 0.304 | 0.529 | 0.129 | 1 | 0.765 |
| 7 | orig. | 0.1258 | 187886 | 47376 | 0.0985 | 434.1 | 0.6455 | 0.1055 | 0.8184 | 113.15 | 32.24 |
|  | [0,1] | 0.509 | 0.201 | 0.101 | 0.547 | 1 | 0.203 | 0.53 | 0 | 0.744 | 0.755 |
| 8 | orig. | 0.2267 | 146692 | 32072 | 0.089 | 405 | 0.465 | 0.0988 | 0.8186 | 87.81 | 29.97 |
|  | [0,1] | 1 | 0.142 | 0.064 | 0.481 | 0.856 | 0.135 | 0.415 | 0.016 | 0.411 | 0.551 |
| 9 | orig. | 0.0566 | 126235 | 22968 | 0.1416 | 329.8 | 0.365 | 0.0894 | 0.8216 | 77.54 | 27.85 |
|  | [0,1] | 0.172 | 0.112 | 0.042 | 0.843 | 0.482 | 0.097 | 0.253 | 0.23 | 0.276 | 0.36 |
| 10 | orig. | 0.0217 | 98996 | 15422 | 0.1568 | 294.3 | 0.2602 | 0.0822 | 0.8251 | 74.68 | 26.31 |
|  | [0,1] | 0.002 | 0.073 | 0.023 | 0.947 | 0.307 | 0.057 | 0.131 | 0.488 | 0.238 | 0.223 |
| 11 | orig. | 0.1089 | 68251 | 9420 | 0.1595 | 289.8 | 0.1642 | 0.0784 | 0.825 | 76.36 | 24.92 |
|  | [0,1] | 0.427 | 0.029 | 0.009 | 0.966 | 0.284 | 0.02 | 0.065 | 0.48 | 0.26 | 0.097 |
| 12 | original | 0.2227 | 47859 | 5880 | 0.1645 | 300.4 | 0.1114 | 0.0746 | 0.8244 | 77.25 | 23.83 |
|  | [0,1] | 0.981 | 0 | 0 | 1 | 0.337 | 0 | 0 | 0.436 | 0.272 | 0 |

**Electricity consumption, number of enterprises, budget expenditures and city size.**

If one considers the dependence of electricity consumption on the size of the city (S1 Fig), then taking into account all Polish cities, an almost proportional relationship is observed. But again – if the analysis is more detailed and examines the contribution of individual groups of cities to this proportionality, a considerable amount of diversity is included. The cities in which the gross domestic product shows super-linear scaling (main text, Figs 2 and 3) exhibit relationships that are closest to clear proportionality. In turn, the cities in which the dependence of the gross domestic product on their size was almost proportional exhibit sublinear scaling here. Are these properties linked? It is likely, as that growth must be associated with an increase in energy consumption.

**Fig S1.** Scaling of electricity consumption in all Polish cities (A) in 2002, (B) in 2012, and the *β* exponent of electricity consumption scaling in relationship to groups of cities.

Again, it is clear that over time the curves connecting the exponent values throughout neurons smooth out; i.e., the value of the differences between groups of cities declines. The biggest quantitative leap in terms of electricity consumption was made in the cities grouped in neuron 12. The analyzed data did not allow, however, the cause of such behavior to be discerned.

The dependence of the number of economic activities on the size of the city (S2 Fig) looks very similar as well – when considering all of the investigated cities, it is close to proportionality. This is worth thinking over, as research into the scaling laws of cities often indicates the super-linear relationships of the attractiveness of large cities. Meanwhile, the activity of its inhabitants is an important feature of the city and does not change in an expected way – it remains proportional to the size of the city for most groups.

**Fig S2.** Scaling of number of enterprises in all Polish cities (A) in 2002, (B) in 2012, and the *β* exponent of number of enterprises in relationship to groups of cities.

An exception is made for cities grouped in neurons 6 and 11 (again), in which the value of the exponent reaches values of $\beta=1.06$ and $\beta=0.88$, respectively. But these results provide no further clarification, showing diverse trends for different types of cities.

The trends depicting the relationships between city budget expenditures and their size are clearly different than the others.
On one hand, the regression line for all cities of all sizes is best fit (S3 Fig) – performed using ordinary least squares minimization – when expressed by a second degree polynomial equation.
On the other hand, any trends disappear for cities grouped in neurons with higher numbers (in the case of Poland, in the direction of smaller cities).

**Fig.S3.** Scaling of number of budget expenditures in all Polish cities (A) in 2002, (B) in 2012, and the *β* exponent of budget expenditures in relationship to groups of cities.

Figure S3, besides the distribution, also shows changes in the value of the exponent during the transition from neuron to neuron. However, the lower value of the exponent is mainly due to the fact that significant uncertainty occurred in determining the trends of the cities grouped into the following neurons. This is better shown in S4 - S6 Figs.

**Fig S4**. Scaling of budget expenditures (2012) of cities grouped in neurons 1-4.

**Fig S5**. Scaling of budget expenditures (2012) of cities grouped in neurons 5-8.

**Fig S6**. Scaling of budget expenditures (2012) of cities grouped in neurons 9-12.

The value of R^2^ for the cities of neuron 12 is so low that it is difficult to speak about any particular trend in the distribution of the relationship between expenses and the size of the city. The city of about 3,000 inhabitants may have expenses of 1.3 million PLN per year, or PLN 2.2 million, or 3.8 million PLN. However, in neuron 1 the trends are better pronounced.

The number of research activities on the subject of the economic effectiveness of cities suggests that checking the postulate of the profitability of urban growth (understood as the balance between socio-economic benefits and the costs of infrastructure and social wealth) seems to remain the center of attention. Thus, it is reasonable to question the accuracy of the scaling laws in anticipation of these features.
Our findings show many interesting phenomena associated with the economy (energy flows) of cities.
We should mention the fact that the variability of income per surface area of the city between groups (neurons) is much greater than the budget expenditures per surface area. The average value of GDP / ha. in cities grouped in neuron 1 is 70 times higher than in cities grouped in neuron 12. But expenditures / ha. for cities in neuron 1 is only 8 times higher than that of cities in neuron No. 12.
Moreover, although the expenditures of cities generally grow with increasing GDP / ha., there are exceptions to this rule: for cities in neuron 8 and in groups from 5 to 3, the average value of expenditure / ha. falls with an increase in GDP / ha.

The variability of the number of enterprises per capita (F7) is also worth exploring.

Ranging from neuron 4 to 8, the average number of enterprises is stabilized and even slightly increases, although the general trend shows that when passing from neurons 1 to 12, along with a reduction in city size, the economic activity of their residents also decreases. This is also related to a significant and disproportionate increase in power consumption (F10). By thoroughly analyzing the variability of individual features, non-linear relationships between the features become visible, similar to the effects of phase transition.

As supplementary material we enclose S7 – S18 Figs, showing relationships between Gross Domestic Product of cities and their population, in each neuron, in year 2002 compared to year 2012 , as an illustration of changes mentioned in the results chapter.

**Fig S7.** Scaling of GDP in cities grouped in neuron no.1 in 2002 (blue) and in 2012 (red).

**Fig S8.** Scaling of GDP in cities grouped in neuron no.2 in 2002 (blue) and in 2012 (red).

**Fig S9.** Scaling of GDP in cities grouped in neuron no.3 in 2002 (blue) and in 2012 (red).

**Fig S10.** Scaling of GDP in cities grouped in neuron no.4 in 2002 (blue) and in 2012 (red).

**Fig S11.** Scaling of GDP in cities grouped in neuron no.5 in 2002 (blue) and in 2012 (red).

**Fig S12.** Scaling of GDP in cities grouped in neuron no.6 in 2002 (blue) and in 2012 (red).

**Fig S13.** Scaling of GDP in cities grouped in neuron no.7 in 2002 (blue) and in 2012 (red).

**Fig S14.** Scaling of GDP in cities grouped in neuron no.8 in 2002 (blue) and in 2012 (red).

**Fig S15.** Scaling of GDP in cities grouped in neuron no.9 in 2002 (blue) and in 2012 (red).

**Fig S16.** Scaling of GDP in cities grouped in neuron no.10 in 2002 (blue) and in 2012 (red).

**Fig S17.** Scaling of GDP in cities grouped in neuron no.11 in 2002 (blue) and in 2012 (red).

**Fig S18.** Scaling of GDP in cities grouped in neuron no.12 in 2002 (blue) and in 2012 (red).

# Supporting information references:

Bendoricchio G, Jørgensen SE. Exergy a goal function of ecosystems dynamic. Ecological Modelling. 1997; 102: 5-15.

Odum H. Self-organization, transformity and information. Science. 1988; 242: 1132-1139.

Patten BC. Network integration of ecological extremal principles: exergy, emergy, power, ascendency, and indirect effects. Ecological Modelling. 1995; 79: 75-84.

Ulanowicz RE. Flow network ascendency and self-organization in living systems. In: Systems and Control Encyclopedia. Singh MG, editor.  Oxford: Pergamon Press; 1987. pp. 1694-1695.

Rutledge RW, Basorre BL, Mulholland RJ. Ecological stability: an information theory viewpoint. J. theor. Biol. 1976; 57: 355-371.

Ulanowicz RE. A phenomenology of evolving networks. Systems Research. 1989; 6: 209-217.

Ulanowicz RE. Network growth and development: Ascendency. In: Complex Ecology: the part-whole relation in ecosystems. Patten BC, Jorgensen SE, editors. New Jersey: Prentice Hall, Englewood Cliffs; 1995. pp. 643-655.

Ulanowicz RE. The balance between adaptability and adaptation. BioSystems. 2002; 64: 13-22.

Bettencourt LMA, Lobo J, Helbing D, Kühnert C, West GB. Growth innovation, scaling and the pace of life in cities. Proc. Natl. Acad. Sci. U.S.A. 2007; 104: 7301-7306.

Jiao Y, Lloyd CR, Wakes SJ. The relationship between total embodied energy and cost of commercial buildings. Energy and Buildings. 2012; 52: 20-27.
